# Absence of both MGME1 and POLG EXO abolishes mtDNA whereas absence of either creates unique mtDNA duplications

**DOI:** 10.1016/j.jbc.2024.107128

**Published:** 2024-03-01

**Authors:** Christian D. Gonzalez, Nadee Nissanka, Derek Van Booven, Anthony J. Griswold, Carlos T. Moraes

**Affiliations:** 1 MSTP and MCDB Programs, University of Miami Miller School of Medicine, Miami, Florida, USA; 2 Department of Neurology, University of Miami Miller School of Medicine, Miami, Florida, USA; 3 John P. Hussman Institute for Human Genomics, University of Miami Miller School of Medicine, Miami, Florida, USA

**Keywords:** mtDNA, mitochondria, MGME1, POLG, duplications, replication, D-loop

## Abstract

Both POLG and MGME1 are needed for mitochondrial DNA (mtDNA) maintenance in animal cells. POLG, the primary replicative polymerase of the mitochondria, has an exonuclease activity (3′→5′) that corrects for the misincorporation of bases. MGME1 serves as an exonuclease (5′→3′), producing ligatable DNA ends. Although both have a critical role in mtDNA replication and elimination of linear fragments, these mechanisms are still not fully understood. Using digital PCR to evaluate and compare mtDNA integrity, we show that *Mgme1* knock out (*Mgme1 KK*) tissue mtDNA is more fragmented than POLG exonuclease–deficient “Mutator” (*Polg MM*) or WT tissue. In addition, next generation sequencing of mutant hearts showed abundant duplications in/nearby the D-loop region and unique 100 bp duplications evenly spaced throughout the genome only in *Mgme1 KK* hearts. However, despite these unique mtDNA features at steady-state, we observed a similar delay in the degradation of mtDNA after an induced double strand DNA break in both *Mgme1 KK* and *Polg MM* models. Lastly, we characterized double mutant (*Polg MM*/*Mgme1 KK*) cells and show that mtDNA cannot be maintained without at least one of these enzymatic activities. We propose a model for the generation of these genomic abnormalities which suggests a role for MGME1 outside of nascent mtDNA end ligation. Our results highlight the role of MGME1 in and outside of the D-loop region during replication, support the involvement of MGME1 in dsDNA degradation, and demonstrate that POLG EXO and MGME1 can partially compensate for each other in maintaining mtDNA.

Maintenance of mitochondrial DNA (mtDNA) relies on a variety of processes, such as mtDNA replication, repair, and nucleotide synthesis, which are aimed at preserving the structural and functional integrity of mtDNA molecules (1, 2). mtDNA maintenance defects, due to mutations in proteins that involved in these processes, are associated with clinically and genetically heterogeneous multisystemic disorders which comprise one of the most common group of inherited metabolic diseases in humans (3). These mutations can lead to either mtDNA depletion or accumulations of aberrant mtDNA molecules (*e.g.* deletions, duplications, rearrangements, or linear fragments) (3). However, how these aberrant mtDNA molecules are generated, propagated, and their role in the pathophysiology of disease is only partially understood.

Polymerase gamma, POLG, is the primary replicative polymerase of the mitochondria. It also harbors a 3′-5′ exonuclease domain, canonically designated as the proofreader. When this domain is mutated in mice, a premature aging phenotype ensues in a model known as the Mutator mouse (4, 5). Mitochondrial genome maintenance exonuclease 1, MGME1, functions as a 5′-3′ exonuclease key to the creation of ligatable ends during replication (6). Knockout of the gene in mice is viable but is associated with inflammatory nephropathy, depletion, and deletions in mtDNA (7, 8). *In vitro*, POLG, and MGME1 form part of the minimal mtDNA replisome (9). Mutations in these enzymes in patients have been associated with the presence of aberrant mtDNA molecules (1). In addition to their role in replication, recent studies have suggested that POLG and MGME1 are also involved in the degradation of linearized mtDNA after double strand breaks (DSBs) (10, 11), although this function for MGME1 is still debated (8).

Although previous studies have detected rearrangements in the mtDNA of POLG exonuclease–deficient (*Polg* Mutator, *Polg MM*) (4, 5) and MGME1-deficient (*Mgme1 KK*) mouse and cell models (7, 12), the structures of these rearrangements and the mechanisms by which they are generated are not fully understood.

We now provide supporting evidence that both MGME1 and POLG EXO are required for the efficient degradation of linearized mtDNA after induced DSBs. We also show that mtDNA fragmentation is related to replication, distance from the origin of replication (O_H_) and type of tissue, with more fragmented mtDNA detected in *Polg MM* and even more strikingly in *Mgme1 KK* than WT animal tissue. Analysis of rearrangement breakpoints indicates that MGME1 has an important role during mtDNA replication in addition to end ligation. Finally, we show that at least one of these replicative exonucleases is required for mtDNA replication and maintenance.

## Results

### Patterns of mtDNA degradation in response to double strand breaks in MGME1 and POLG exonuclease mutants

**Figure 1:**
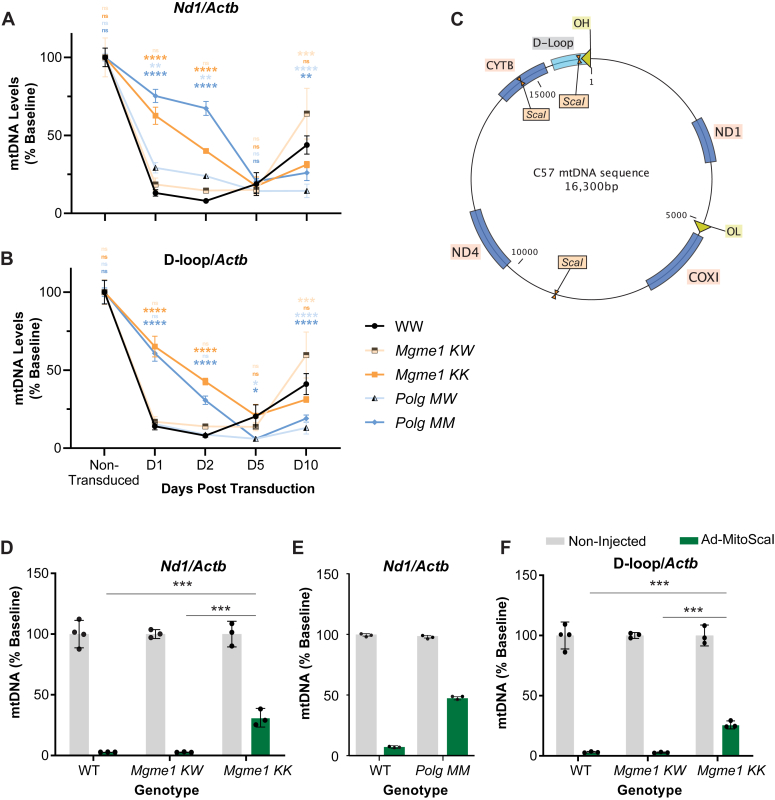
**MtDNA rate of elimination after double strand breaks.***A*, C57 mtDNA map showing relevant regions of interest/targets for the assays. *B*–*E*, mtDNA levels are reported as a % Baseline derived from the standardized ΔΔCT quantification method for qPCR using the non-infected samples as a reference (100% mtDNA). Points on scatter plots (*B* and *C*) are an average (n = 3), and error bars are SD. Bar graphs (*D* and *F*) show the mean n = 3 to 4 animals or n = 1 showing three technical replicates (*E*). A two-way ANOVA was performed to analyze the effect of genotype and time elapsed post-transduction on mtDNA levels. Multiple comparison analysis with Tukey correction was used to determine the statistically significant differences depicted on the graphs. (ns = not significant, ∗*p* < 0.05, ∗∗*p* < 0.01, ∗∗∗*p* < 0.005, ∗∗∗∗*p* < 0.0001). mtDNA, mitochondrial DNA; qPCR, quantitative PCR.

We and others have previously shown that eliminating POLG exonuclease (EXO) function results in delayed degradation of linear mtDNA fragments in mice and cell models after induced double strand breaks (10, 11). Similar findings were reported for *Mgme1 KK* cells (11). However, other studies suggested that MGME1 is not involved in mtDNA degradation following DSB (8, 13). Thus, to simultaneously evaluate the degradation of linearized mtDNA (after induced double strand breaks) by these exonucleases, we transduced WT, *Mgme1 KK, Mgme1KW, Polg* MW, and *Polg MM* mouse pulmonary fibroblasts with a recombinant adenovirus expressing a mitochondrial-targeted *Sca*I restriction endonuclease (rAd-mito*Sca*I-HA). These cells were then analyzed 1-, 2-, 5-, and 10-days post transduction. Using quantitative PCR (qPCR), we determined relative mtDNA levels (*Nd1* and D-loop regions) to a nuclear DNA reference gene (*Actb*). The relevant regions of mtDNA which were targeted in this study (D-loop*, Cytb, Nd4, Cox1*, and *Nd1*) are shown in Figure 1*C*. We observed that cells lacking functional MGME1 or POLG EXO had a delayed reduction in mtDNA (intact or fragmented) in days 1 and 2 post-mito*Sca*I transductions when compared to the WT controls. WT cells experienced the most rapid decline in mtDNA levels, reaching their minimum levels of mtDNA on day 2, whereas mutant cells exhibited a slower decrease, reaching their minimum levels of mtDNA on day 5 (Fig. 1, *A* and *B*). MtDNA from heterozygote lines (*Polg MW* and *Mgme1 KW)* was eliminated at the same rate as mtDNA from WT cells (Figs. 1, *A* and *B* and S1, *C* and *D*). The initial rate (days 1–2) of mtDNA degradation in *Mgme1 KK* v *Polg MM* cells differed slightly (∼10%) as measured by *Nd1/Actin* (Fig. S1*C*). Due to low mtDNA levels in *Mgme1KK*, the % changes from the baseline are greater than in cells with higher mtDNA levels like *Polg MM* samples. Despite this, the difference in the rate of degradation as measured by *Nd1/Actin* between either mutant and control cells was considerably greater (∼50%) (Fig. S1*C*). We find that when the relative mtDNA levels are similar at baseline, as when measured by D-loop/*Actin* (because of 7S DNA accumulation in *Mgme1 KK*), the rate of mtDNA degradation for both *Polg MM* and *Mgme1 KK* was nearly identical (7, 8, 12) (Fig. S1*B*). These data, which provide a direct comparison between these two models, shows that the rate of mtDNA degradation after DSB is similar when either MGME1 or POLG EXO activities are impaired.

To determine if these same defects were observed *in vivo*, we injected (1 month old) C57 WT, *Mgme1KW* ,and *Mgme1 KK* mice with rAd-Mito*Sca*I retro-orbitally (i.v.) and collected mouse liver 5 days after infection. Expression of mito*Sca*I in the liver was confirmed by Western blotting and immunohistochemistry (Fig. S2, *A* and *B*). Liver DNA was then analyzed by qPCR using the same *Nd1*, D-Loop, and *Actb* assays mentioned above (Fig. S1, *A* and *E* and *F*). Five days post infection with rAd-Mito*Sca*I, C57WT and *Mgme1KW* mice both had a marked decrease in mtDNA levels (*Nd1* and D-Loop), nearly to 0%, while *Mgme1 KK* kept around 30% of their baseline mtDNA levels (Fig. 1, *D* and *F*) which fell in line with the results seen in cultured cells. The same experimental conditions were previously used with the *Polg MM* model (10). We included the POLG data in Figure 1*E* for comparison, where a similar delay in mtDNA elimination was observed in the liver of POLG mutator mice after induced DSB.

### Characterization of mtDNA fragmentation patterns by 2D plot analysis of digital PCR

We next characterized the fragmentation features of mtDNA in the WT, *Polg MM*, and *Mgme1 KK* models without induced DSB. To do this, we implemented both a 1-dimension (1D) and a 2-dimensional (2D) plot analysis of digital PCR (dPCR) data. During dPCR, one sample is segregated into many thousand partitions which undergo parallel amplification, and the analysis software can plot partitions in 1 dimension (which is used for copy number determination, Fig. S3*A*) or in 2 dimensions to determine if two independent targets (in a multiplex reaction) are in the same molecule. Molecules that contain two targets would result in double positive partitions (DPPs) whereas molecules containing only one target would result in single positive partitions (SPPs) (Fig. S3*B*).

To verify that this approach could adequately quantify fragmented molecules in a mixture of molecules, we built a 16.6 kb circular DNA plasmid, modeling a circular mtDNA molecule, which contained specific diametrically located sequences (TAG1 and TAG2, which are uniquely generated sequences), as well as mouse *Nd1* and *Nd4* sequences (detectable by our prior mentioned assays) and used restriction enzymes to generate linearized (*Xho*I digested) and fragmented molecules (*Sma*I digested) (Fig. S3*C*). Mixing circular, linear, and fragmented molecules in various ratios resulted in expected and proportional changes in 2D % Positive Partitions (2D %PP, Figs. S3*D* and S4*A*). By analyzing the dPCR data in 1D and 2D, we obtained complementary data, which provided insight into the structure of these molecules (exemplified in Fig. S4*B*).

### MGME1 and POLG EXO prevent the accumulation of fragmented mtDNA

**Figure 2:**
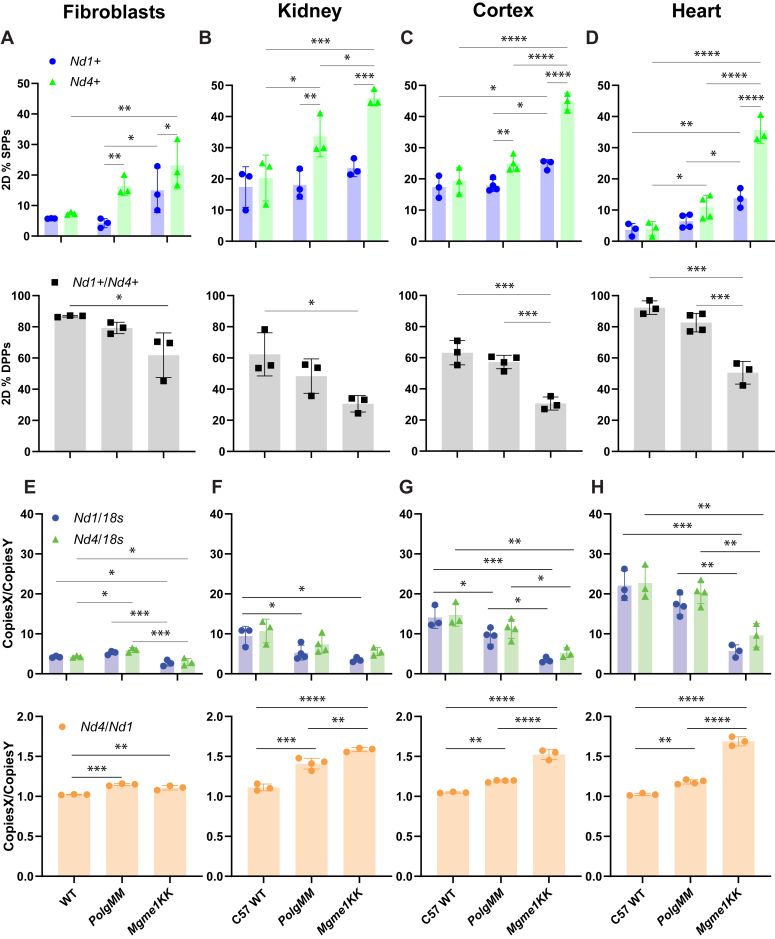
**2D and 1D dPCR analysis of *Mgme1 KK* and *Polg MM* fibroblasts and tissues.***A*–*D*, the fragmentation profiles, that is, the 2D %PP of the *Nd1+* and *Nd4+* SPPs (*top row*) and *Nd1+*/*Nd4+* DPPs (*bottom row*) were calculated *via* 2D plot dPCR analysis (Fig. S3). *E*–*H*, copy ratios were generated *via* dPCR 1D analysis between mtDNA targets, *Nd1* or *Nd4,* and genomic target *18s* (*top row*), and between *Nd1* and *Nd4* (*bottom row*). For fibroblasts, we used three different passage numbers per cell line (n = 3). For animal tissues, there are 3 to 4 different animals per genotype. Each point represents one of the passage numbers or an individual animal; bars represent the average of all passages, or animals, error bars are SD. Two-way ANOVAs were performed to compare the effect of genotype and mtDNA target on 2D %PP for SPPs (*top row*). One-way ANOVAs were performed to compare the effect of genotype on 2D%PP for DPPs (*bottom row*). Multiple comparison analysis with Tukey correction was used to determine the statistically significant differences depicted on the graphs. (∗*p* < 0.05, ∗∗*p* < 0.01, ∗∗∗*p* < 0.005, ∗∗∗∗*p* < 0.0001). dPCR, digital PCR; DPP, double positive partition; mtDNA, mitochondrial DNA; SPP, single positive partition.

Once validated, we used the 2D dPCR method to characterize the fragmentation profile of our biological samples. We first evaluated the fibroblast models using two assays targeted to diametrically opposed targets on the mtDNA molecule, *Nd1* and *Nd4*. *Nd4* was chosen as it is part of the linear “major arc” fragment, an approximate 11 kb fragment from O_H_ to O_L_, previously found to be present in both *Polg MM* and *Mgme1 KK* (5, 7). We observed that *Mgme1 KK* fibroblasts have the lowest *Nd1*+/*Nd4*+ DPPs (proxy for intact mtDNA) than WT and *Polg MM* fibroblasts, with a corresponding increase in *Nd4*+ SPPs (proxy for fragmented mtDNA) compared to WT fibroblasts. There was also a trend for lesser *Nd1*+/*Nd4+* DPPs and greater *Nd4*+ SPPs for *Polg MM* than WT fibroblasts (Fig. 2*A*). There was a clear trend toward having more *Nd4+* SPPs than *Nd1+* SPPs in the mutant cells, which could be in part explained by the presence of the “major arc” linear fragment (Fig. 2*A*).

We then examined the fragmentation profile of the tissues of aged C57WT (17M), *Polg* MM (12M), and *Mgme1 KK* animals (15M). We found consistently less *Nd1*+/*Nd4*+ DPPs and more *Nd1*+ and/or *Nd4*+ SPPs in kidney, cortex, and heart of *Mgme1 KK* animals compared to C57WT and *Polg MM* tissue (Fig. 2, *B*–*D*). In addition, there was a consistently large difference between *Nd1*+ and *Nd4*+ SPPs in all tissues of *Mgme1 KK* animals, with greater *Nd4*+ SPPs (Fig. 2, *B*–*D*). In *Polg MM* animals, the difference between these two was not as striking, yet still higher than C57WT, and varied according to tissue type, with kidney having the greatest difference between SPPs (Fig. 2, *B*–*D*). As expected, there was no difference between the *Nd1*+ and/or *Nd4*+ SPPs for WT fibroblasts (Fig. 2*A*) or C57WT mouse tissue (Fig. 2, *B*–*D*). When using biological samples (as opposed to our model molecule), we consistently found considerable levels of SPPs even in controls, which likely corresponds to a mixture of replication intermediates, small percentage of fragmentation, and assay-related noise.

In the *Cytb*-*CoxI* paired assay, designed to measure linked targets in the reported major arc fragment that accumulates in *MgmeI KK* and *Polg MM* animals, we noted less *Cytb*+/*CoxI*+ DPPs in *Mgme1 KK* tissues compared to both C57WT and *Polg MM* animals (Fig. S5). In agreement, there was greater *Cytb+* and/or *CoxI+* SPPs in *Mgme1 KK* animals when compared to C57WT and *Polg MM* animals as well as significant differences between *Cytb*+ and *CoxI*+ SPPs in mutant tissues (Fig. S5). For the *Cytb*-*Nd4* paired assay, targets that are even closer in the reported major arc fragment, the results for cortex and heart were similar to the *Cytb*-*CoxI* paired assay (Fig. S6). All these analyses showed that *Polg MM* and, to an even higher degree, *Mgme1 KK* have relatively high levels of fragmented mtDNA compared to WT control and that there are many types of fragments in addition to the previously described “major arc” linear fragment. Interestingly, the most abundant fragments are closer to the origin of H-strand replication (O_H_), as discussed in more detail below.

### MGME1 and POLG EXO prevent mtDNA segment specific copy number imbalance

**Figure 3:**
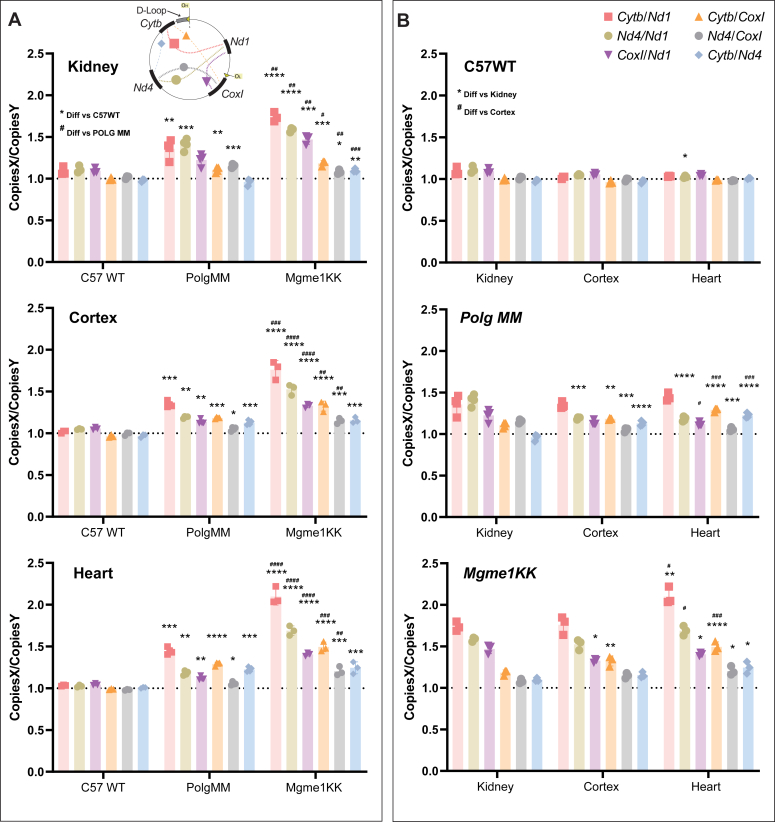
**Ratios of copy number of various mtDNA regions in different genotypes and tissues.** The 1D dPCR copy ratios presented are grouped by tissue type (*A*) or by genotype (*B*). The inset on *top* of the *left panel* illustrates the two regions used for the ratios. mtDNA, mitochondrial DNA; dPCR, digital PCR.

In addition to exploring the fragmentation profile of our cell and tissue models through the described analysis derived from 2D dPCR plots, we also examined the absolute mtDNA copy numbers through 1D dPCR analysis. To get these measurements, we used the same assays described above, along with an *18s* nuclear DNA reference gene assay. In doing so, we can report the number of copies of a target in the sample and create the ratio of any two targets as Copies X/Copies Y. For example, our data shows that WT cells have ∼4 copies of *Nd1* for every 1 copy of *18s* (Fig. 2*E*) in WT cells. Copy ratios containing *18s* allow for the comparison of the mtDNA levels between different samples (Fig. 2, *E*–*H*, Top Row), while ratios between the different mitochondrial DNA targets will show regional copy imbalances found within one sample (Fig. 2, *E*–*H*, Bottom Row). Using this approach, we observed that *Mgme1 KK* tissue and fibroblasts have a decrease in mtDNA levels (Fig. 2, *E*–*H*, Top Row), as previously reported (7, 8, 12), a finding which we also verified by qPCR (Fig. S1*A*). We also show that both *Polg MM* and *Mgme1 KK* tissues and fibroblasts have more *Nd4* than *Nd1* copies (∼10–50%) and that *Mgme1 KK* tissue has a greater *Nd4* to *Nd1* copy imbalance than *Polg MM* tissue (Fig. 2, *E*–*H*, Bottom Row). Our *Mgme1 KK* animals show a much greater accumulation of *Nd4* to *Nd1* than cells in culture. Interestingly, and in agreement with the 2D analyses, we found a step wise decrease of copies the further downstream the target sequence was from the origin of replication of the heavy strand (O_H_) (Fig. 3).

As a summary of the fragmentation analyses: (1) MtDNA fragmentation varied between tissues; (2) replicative exonuclease deficiencies lead to mtDNA regional copy imbalances in all tissues; (3) lack of functional MGME1 results in high mtDNA regional copy imbalances, most strikingly in the heart; (4) lack of functional POLG exonuclease results in milder mtDNA regional copy imbalances; and (5) the distance from O_H_ is related to the relative abundance of mtDNA fragments.

### Sequence coverage steadily declines downstream of O_H_ in *Mgme1 KK* and *Polg MM* heart mtDNA

Having observed the fragmentation and regional copy imbalance in the mtDNA of mutant model cells and animals as reported by our dPCR experiments, we went on to acquire the mtDNA sequence by NGS from the hearts of C57WT, *Polg MM*, and *Mgme1 KK*. As expected, the single nucleotide variation (SNV) of each position on the mtDNA was highest for *Polg MM* heart since the proofreading activity of POLG EXO is absent. The SNV was remarkably similar for C57WT and *Mgme1 KK* animals (Fig. S7*A*). We also found that the sequence coverage of mtDNA for *Mgme1 KK* and *Polg MM* hearts, on average, decreases downstream of O_H_ (Fig. S7*B*), with a much more striking coverage loss in *Mgme1 KK* heart mtDNA. This coverage loss is in line with the results of our dPCR assays (Fig. 3) and a previous report (7). Interestingly, there is a rise in coverage for both *Mgme1 KK* and *Polg MM* between O_H_ and position 15,720 bp, after which there is a decline in coverage for both *Mgme1 KK* and *Polg MM* mtDNA, with a precipitous drop in coverage for the *Mgme1 KK* sample (around the termination associated sequence1-tRNA^Pro^ [TAS1-*trnP*] region) and a more gradual decline observed in the *Polg MM* sample (Fig. S7*C*). Part of this peak is likely a reflection of 7S DNA accumulation; however, the uneven shape of the coverage in both *Mgme1 KK* and *Polg MM* heart mtDNA indicates that it also reflects other D-loop abnormalities.

### NGS reveals unique duplications in *Mgme1 KK* and *Polg MM* heart mtDNA

Mitochondrial Structural Alterations (MitoSALT) is a computational analysis tool designed to identify and quantify deletion and duplication breakpoints in mtDNA. Such sequences are only found in abnormal mtDNA molecules. The determination of whether the breakpoint is associated with a deletion or a duplication is based on the presence of O_H_ (origin of replication, which is required for mtDNA replication) in the putative recombinant molecule. If O_H_ is missing, then the breakpoint is flagged as part of a duplication. This methodology has been described elsewhere (14).

**Figure 4:**
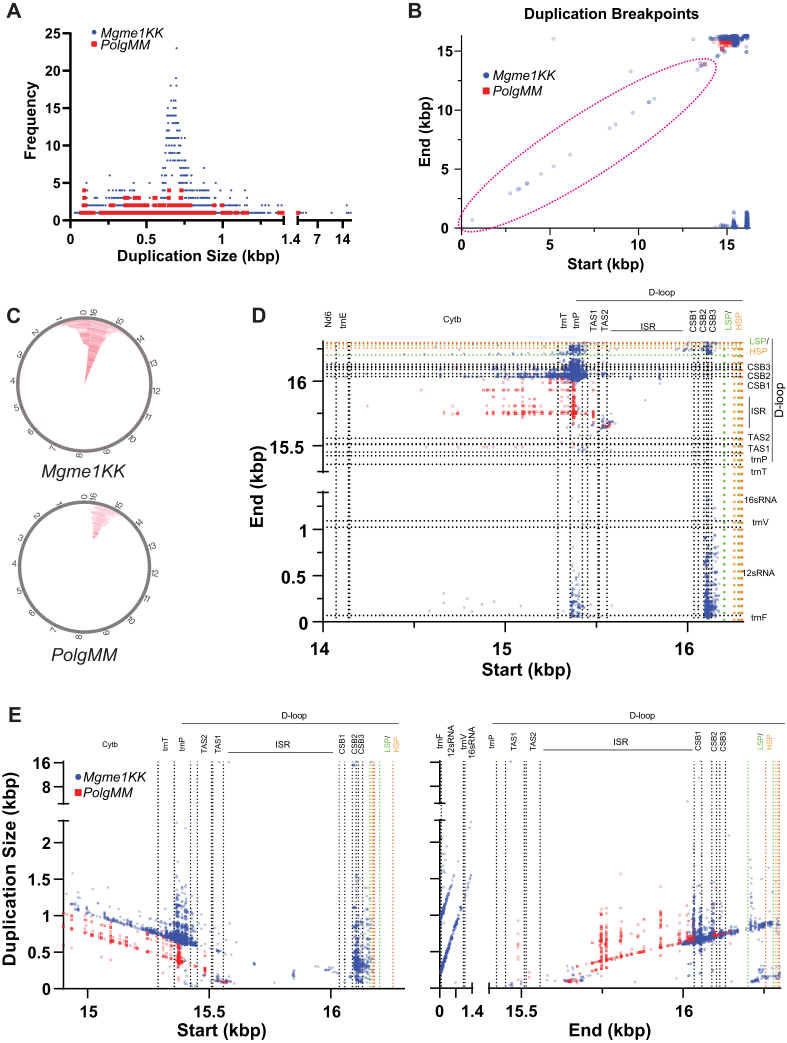
**mtDNA breakpoint mapping and duplication size analysis.***A*, frequency of duplication sizes found in *Polg MM* and *Mgme1 KK* heart mtDNA. *B*, mapping of mtDNA breakpoint start and end sequences superimposed onto a map of the mtDNA molecule by position (14, 15, 16, 17). *C*, mtDNA duplications plots as predicted by mitoSALT analysis pipeline. *Red lines* indicate duplicated regions. *D*, mapping of most frequent mtDNA breakpoints. *E*, duplications show that duplication size is directly proportional to start and end breakpoint position. N = 1 animal heart WGS for both *Mgme1 KK* and *Polg MM*; C57WT heart mtDNA had no breakpoints detected using mitoSALT analysis. Each dot is a separate sequence; translucency was set at 40%, so areas that are more heavily colored are due to high density of reads. mtDNA, mitochondrial DNA; WGS, whole genome sequencing.

Using MitoSALT, we determined that both *Polg MM* and *Mgme1 KK* heart mtDNA samples have an array of duplications (Fig. 4*C*), with *Mgme1 KK* heart mtDNA having particularly high levels. Previous analyses have identified similar duplications in these models (14) but have not analyzed the breakpoint regions in detail. We found that duplications for these models ranged in size from a few nucleotides to almost the complete mtDNA, but the majority between 0.5 to 1 Kbp (Fig. 4*A*). Most breakpoints were found near/within the control region, but a series of breakpoints unique to *Mgme1 KK* heart mtDNA was found along the length of the mtDNA molecule and indicate the presence of similarly sized ∼100 bp duplication (Fig. 4*B*, red dashed circle). Overall, *Mgme1 KK* breakpoints define larger duplications on average (by ∼300 bp) than *Polg MM* breakpoints (Fig. 4*A*). We found that *Mgme1 KK* mtDNA had a high frequency of ∼690 bp duplications while no such peak was observed in *Polg MM* mtDNA, which displays a flat distribution of duplication sizes (˜100 bp–˜1000 bp; Fig. 4*A*). The largest duplication found in *Polg MM* mtDNA is 1565 bp, while the largest duplication in *Mgme1 KK* is 16,248 bp, nearly the full length of mtDNA (Fig. 4, *A* and *E*).

Both *Mgme1 KK* and *Polg MM* mtDNA show duplications whose sizes are directly proportional to start or end breakpoint positions (Fig. 4*E*). This continuous spectrum of duplication sizes suggests that mtDNA replication (3′ extension or 5′ processing) is involved in the generation of these aberrant species. Additionally, some of the hotspot positions were associated with a range of duplication sizes (*e.g.*, *trnP*, conserved sequence block (CSB) 1 to 3). This suggests that either start or end sequence hotspots function as anchors for duplication formation (Fig. 4*E*).

Mapping the breakpoints, we defined seven major distinct groups of duplications, characterized by their start/end position (Fig. 4, *B* and *D*, Table S1). We found that *trnP* and CSB2-3 (where O_H_ is located) regions are hotspots for the start of breakpoints, while CSB1-3, *12sRNA*, and HSP/LSP regions are hotspots for the end of breakpoints in *Mgme1 KK* heart mtDNA (Fig. 4, *D* and *E*). The *trnP* region is also a hotspot for the start of breakpoints in *Polg MM* heart mtDNA, but the end sequences of these breakpoints are found exclusively in a region between the TAS and CSB1, which we refer to as the intersequence region (ISR).

**Figure 5:**
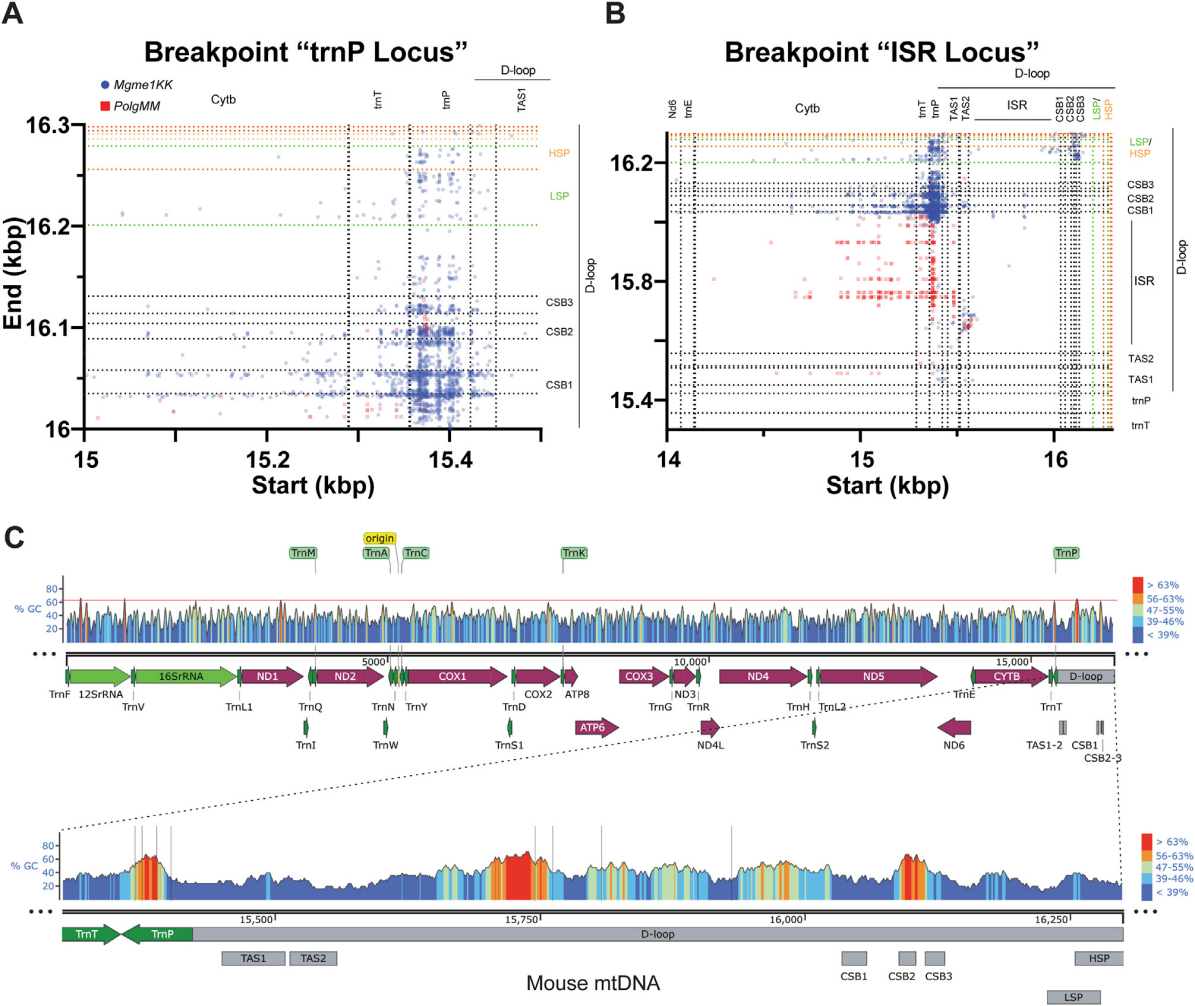
***Mgme1 KK* and *Polg MM* generate unique breakpoints/duplications.***A* and *B*, zoomed in (higher resolution) maps reveal areas of interest within breakpoint hotspots. *C*, mouse mtDNA sequence GC content analysis, with a heat color gradient to highlight GC-rich areas. The *lower* panel zoom-in on the control region. *Black vertical* lines bring attention to the start/end sequences of breakpoints of interest. mtDNA, mitochondrial DNA.

A closer look at the mapped regions showed that breakpoints not only group to specific mtDNA regions but also to specific nucleotide positions within these regions (Fig. 5). *Mgme1 KK* breakpoints starting within *trnP* are localized mostly to 3 sites, 15368, 15388, and 15403 bp (Fig. 5*A*), which altogether surround a GC-rich region (Fig. 5*C*). *Polg MM* mtDNA breakpoints starting within *trnP* grouped to position 15373 bp, another GC-rich region (Fig. 5, *B* and *C*), and breakpoints ending in the ISR localize predominantly to 4 sites, 15746, 15763, 15808, 15931 bp (Fig. 5*B*), none of which are GC-rich, but are found before a large GC-rich segment from 15703 to 15577 bp (Fig. 5*C*). While duplications start anywhere up/downstream of *trnP* for both *Mgme1 KK* and *Polg MM*, duplications for *Polg MM* predominantly end downstream of CSB1, while *Mgme1 KK* duplications predominantly end upstream of CSB1 (Fig. 5, *A* and *B*). Analysis of the breakpoint regions showed the presence of microhomologies in the sequences involved in the rearrangements, mostly in the form of perfect or imperfect direct repeats ranging from 2 to 14 bp (Table S2).

In summary, the NGS/mitoSALT showed that: (1) *Polg MM* and, to a higher degree, *Mgme1 KK* heart mtDNA showed reduced mtDNA sequence coverage downstream of O_H_. (2) *Polg MM* and *Mgme1 KK* heart mtDNA showed distinct loci of breakpoints mainly in the control region of mtDNA. (3) The size of mtDNA duplications is relatively constant (˜100 bp) in areas outside of the control region found exclusively in *Mgme1 KK*. (4) *Mgme1 KK* duplications are overall larger than *Polg MM* duplications and (5) the position-dependent size of a substantial subset of these duplications suggest that the mechanism of formation is related to mtDNA replication.

### At least one replicative exonuclease is required for mtDNA replication

**Figure 6:**
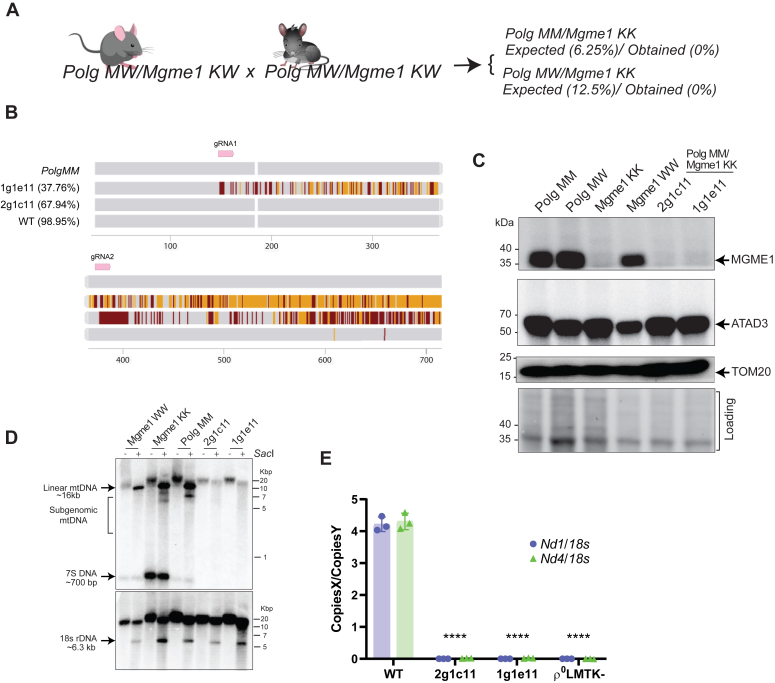
**mtDNA cannot be maintained in *Polg MM*/*Mgme1 KK* double mutant cells.***A*, expected frequency of pairings according to Mendelian probabilities. *B*, sanger sequencing alignment traces of the clones 2g1c11 (from gRNA2) and 1g1e11 (from gRNA1) obtained after RNP-CRISPR. *Orange* means there is no call, likely due to multiple alleles present, while *dark red* means there is a variant call. The pairwise identity is noted (%). *C*, Western blot shows the absence of MGME1 in clones derived from *Polg MM* fibroblasts. *D*, Southern blot of cell models. DNA was post-digested with *Sac*I HF to linearize DNA. In the *top* blot, DNA was labeled with a probe spanning the D-loop region of mtDNA. In the *bottom* blot, DNA was labeled with a *18s* DNA probe to serve as loading control. *E*, copy number analysis *via* dPCR was done on the double mutant clones 2g1c11 and 1g1c11 (*Polg MM/Mgme1 KK*) as well as a control cell line ρ^0^ LMTK- cells which are devoid of mtDNA. Different passages of the cell lines were used as replicates (n = 3). A one-way ANOVA was performed to compare the effect of cell genotype on *Nd1*/*18s* and *Nd4/18s*. Multiple comparison analysis with Tukey correction was done to determine the statistically significant differences depicted on the graphs. (∗∗∗∗*p* < 0.0001). dPCR, digital PCR; mtDNA, mitochondrial DNA.

Although mtDNA changes associated with defects in the nuclease activity in either enzyme have been analyzed, the consequences of lacking both activities are unknown. Therefore, we attempted to generate mice and cell models deficient in both POLG EXO and MGME1. We crossed a variety of *Polg* and *Mgme1* mutant mice with the goal of generating *Polg MM*/*Mgme1 KK* double mutant animals. However, after attempting multiple crosses and analyzing over 100+ pups, we were unable to generate a single *Polg MM*/*Mgme1 KK* mutant mouse (Figs. 6*A* and S8). Interestingly, there were also no *Polg MW/Mgme1 KK* animals in any of the crossings and *Polg MM*/*Mgme1KW* animals were generated at less than that expected frequencies (Figs. 6*A*, S8). Efforts to isolate double mutant mouse embryonic fibroblasts from embryos isolated from pregnant females (E7-10) were also not successful, as none of the mouse embryonic fibroblasts were double mutant.

In parallel, we used RNP-CRISPR Cas9 to knockout *Mgme1* in *Polg MM* fibroblasts. Using this approach, we obtained several clone candidates that showed out of frame mutations in the exon 2 of *Mgme1*. Figure 6*B* and Fig. S9 show the alignment of two of these clones (Clones 2g1c11 and 1g1e11) to the *PolgMM* parental cell line. These clones were then screened for the presence of MGME1 *via* Western blot. Clones 2g1c11 and 1g1e11 both lacked MGME1 protein, while having normal or increased levels of other mitochondrial markers (ATAD3 and TOM20, Fig. 6*C*). We then attempted to analyze the mtDNA in these clones, but we found that their mtDNA levels were below detection (Fig. 6*E*). The dPCR data was confirmed by Southern blot (Fig. 6*D*). Thus, we concluded that at least one replicative exonuclease is required for mtDNA replication/maintenance.

## Discussion

The last 40 years of mitochondrial research has shed light on several factors that regulate mtDNA maintenance; however key questions remain unanswered. Currently, there is no consensus on the presence and mechanisms of mammalian mtDNA repair, particularly after DSB. However, recent reports suggest that the mtDNA replisome can mediate recombination of free DNA ends (15). Thus, the action of two replisome exonucleases, POLG EXO and MGME1, in eliminating broken mtDNA after DSB and their role in mtDNA maintenance has come into focus (7, 8, 10, 11). In the present study, we show that POLG exonuclease and MGME1 are both necessary for the efficient degradation of mtDNA after induced double strand breaks. Interestingly, in the absence of one or the other, a series of mtDNA abnormalities (fragmentation and rearrangements) occur. The molecular features of the aberrant molecules found in *Mgme1 KK* tissue suggest a role in mtDNA homeostasis that goes beyond its previously suggested role in in the creation of ligatable ends at the end of replication. Moreover, even though mtDNA is present when one of these replicative exonucleases is missing, mtDNA is not maintained when both are absent, providing *in vivo* evidence for their coordinated roles in mtDNA replication and demonstrating that at least one of these replicative exonucleases is necessary to maintain mtDNA.

### The functions of MGME1 and POLG EXO after DSB

Our data support the concept that MGME1 and POLG exonuclease are key players in the degradation of linearized mtDNA resulting from DSBs (10, 11). Interestingly, the combined action of POLG EXO and MGME1 in degrading linearized mtDNA after DSBs is more efficient than the action of either one alone, as evidenced by the impaired daily rate of change when either activity is missing. Nonetheless, despite deficiencies in either replicative exonuclease, mtDNA is eventually degraded, suggesting that the remaining exonuclease (MGME1 or POLG EXO) functions alone or that other less efficient mechanisms degrade mtDNA at a slower rate. Mitochondria possess several nucleases which could have this secondary fragment elimination ability (16); however knocking them down has not shown a major effect on fragmented mtDNA elimination (13), suggesting that POLG EXO and MGME1 are the main players on the elimination of linear mtDNA fragments. Because cells lacking both activities lack mtDNA, mtDNA degradation in a double mutant model could not be assessed.

### The functions of MGME1 and POLG EXO in mtDNA replication/maintenance

Even though both POLG EXO and MGME1 enzymes are involved in degradation of linear mtDNA fragments after DSBs, it is worth noting that the mtDNA levels are different for *Polg MM* and *Mgme1 KK* models. This reflects the different activities for each of these enzymes during replication or perhaps in other repair contexts outside of DSBs. Our current study and others (6, 7, 8, 12) showed that knocking out *Mgme1* results in pronounced mtDNA depletion, large increases in 7S DNA levels, and accumulation of mtDNA rearrangements (6, 7, 11, 17) and that *Polg MM* models while not showing mtDNA depletion, or similar increases in 7S DNA levels, accumulate high levels of mtDNA mutations as well as mtDNA rearrangements, aberrant control region multimers, and mtDNA deletions (5, 10, 18, 19). In both models, the presence of a subgenomic fragment spanning the major arc region has been reported (5, 7, 8, 10, 20). While previous studies have provided some insights into the nature of these aberrant mtDNAs, quantification and characterization of these species has proved difficult. Sequencing techniques alone rely on fragmentation of the genome that interferes with determination of mtDNA integrity. Traditional techniques like 2D AGE and Southern blot often rely on using high amounts of DNA and when it comes to characterization of fragments and structures, rely on uniformly sized DNA molecules for detection. In this study, we took advantage of 1D/2D dPCR analysis alongside NGS and mitoSALT analysis to analyze novel quantitative and qualitative differences in mtDNA features of *Mgme1 KK* and *Polg MM* models.

Because of the published evidence showing that mtDNA rearrangements in postmitotic tissues increase with age (21, 22, 23), we studied mice as old as possible considering the shortened lifespan of mutants (12–15 months old). Although we cannot rule out that some rearrangements may be selected against, we reasoned that older mice would likely show the highest levels of heteroplasmic abnormal mtDNA. Overall, our cell and animal models showed that absence of MGME1 results in higher levels of mtDNA fragmentation than the absence of POLG EXO, although both have more mtDNA fragmentation than C57WT. Interestingly, our data suggests that there are more fragments produced in addition to the previously reported major arc fragment. Why is the mtDNA fragmented in these mutants? We propose that two factors contribute to it: 1) Replication is impaired, which can lead to aborted intermediates. This may occur more often early in replication, which would explain the O_H_-distance dependence of fragments, observed both by dPCR and NGS; 2) such fragments would not be efficiently eliminated by the individual nucleases.

**Figure 7:**
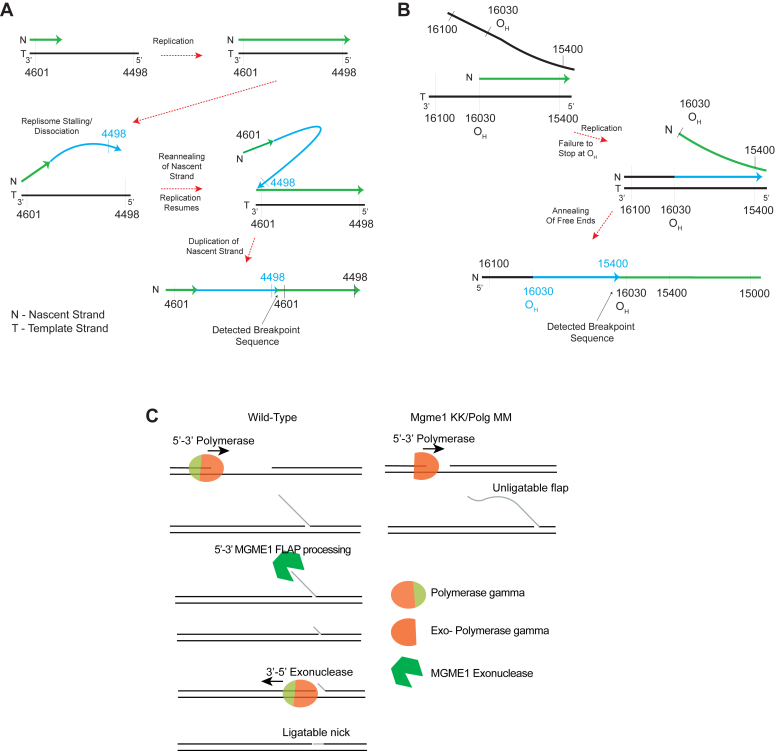
**Proposed mechanism for aberrant mtDNA maintenance.***A*, proposed model for the formation of non-control region 100 bp duplications. Replication slippage/dissociation of replisome could be favored in *Mgme1* mutants for regions of ˜100 bp, which could reanneal and form duplications, as observed in *Mgme1 KK* heart. *B*, proposed model for the formation of duplications in the D-loop region in *Mgme1 KK* and *Polg MM* mutants. As described *in vitro*, MGME1 and POLG EXO act synergistically at the end of replication, generating ligatable ends. When these factors are missing, abnormal displacements of nascent strand when the replisome fails to stop at O_H_ results in atypical ligation of these ends creating the duplications detailed in this study. *C*, proposed model for the lack of mtDNA replication in the absence of both MGME1 and POLG EXO. Both enzymes have a role in the creation of ligatable nascent mtDNA ends at the end of replication. Absence of MGME1 or POLG EXO still allows for mtDNA replication. However, absence of both results in mtDNA depletion likely because of non ligatable flaps. mtDNA, mitochondrial DNA.

NGS/mitoSALT analysis revealed that *Mgme1KK* heart mtDNA has much higher levels of duplications than *Polg MM* heart mtDNA. Evaluating the breakpoints associated with these duplications revealed six major groups of breakpoints for *Mgme1 KK* tissue while only ome major group of breakpoints for *Polg MM* heart mtDNA was identified, highlighting the distinct roles of both proteins in preventing abnormal mtDNA rearrangements, end processing, and ligation. Of particular interest is the group of breakpoints found in *Mgme1KK heart mtDNA* that are associated with the ∼100 bp duplications that occur continuously along the length of the mtDNA molecule. Based on the positions and directionality of the breakpoint start and end sequences, we propose that these duplications result from replication slippage secondary to replisome dissociation, mediated by microhomologies resulting in reannealing and duplication formation (Fig. 7*A*). If the replisome fails to re-associate, the nascent strand will likely denature from the template strand, resulting in mtDNA fragments.

Our data on mtDNA rearrangements suggests that MGME1 can directly or indirectly prevent the dissociation of the replisome from template DNA. Although speculative, this potential mechanism would support the previous reports which suggest that the 7S DNA accumulates through continuous abortive replications due to lack of MGME1 (8) but also implies that MGME1 has a function during replication and not only at the end of replication. On the other hand, besides proofreading, the duplication breakpoint features suggest that POLG EXO is involved mostly in processing mtDNA for end ligation at the end of replication.

Several studies have proposed diverse models of mtDNA recombination (2, 24, 25, 26). Both *Mgme1KK* and *PolgMM* heart mtDNA samples showed breakpoints that start in the tRNA Proline gene (*trnP*) and end in upstream sequences (ISR, CSB1-3, HSP/LSP, *12sRNA*). Most, if not all these breakpoints showed the presence of microhomologies mediating the breakpoint formation. It has been previously reported that POLG EXO disengages the strand displacement function of POLG (27). Here, we observed that in *Mgme1KK* tissue, there were duplications (16,248 bp) that were nearly the size of the entire mtDNA molecule. Considering these observations, we propose that these duplications occur through the ligation of atypical DNA ends created when the replisome fails to stop at the O_H_, resulting in strand displacement of the 5′ end of the nascent strand by the 3′ end of the same strand nearing completion of replication (Fig. 7*B*). In *Mgme1KK* tissue, the inability of MGME1 to digest the 5′ end of the nascent strand could lead to breakpoints ending in HSP/LSP or CSB regions, which are not found in *PolgMM* tissue. Lastly, only *Mgme1KK* heart mtDNA has breakpoints which start at *trnP* or CSB and end along a range of endpoints in *12sRNA*. These groups of breakpoints do not fit either of the proposed mechanisms (replication slippage or strand displacement). It is likely that these breakpoints occur due to recombination or some other uncharacterized mechanism. According to these observations, MGME1 and not POLG EXO has a role in preventing these rearrangements. *Polg MM* mtDNA has no breakpoints along the length of the mtDNA molecule or those that suggest alternate mechanisms of deletion formation. It is evident then that *Polg MM* mtDNA shows less regional copy imbalances, lower amounts of mtDNA fragmentation, and lower levels of duplications than *Mgme1 KK* mtDNA.

What makes a specific mtDNA region a hotspot for rearrangements? The CSB2-3 regions contain O_H_, a GC-rich region which has been described to form G-quadruplex structures (1). These regions have been reported to have a role in replication stalling (28, 29, 30) and in DNA recombination (31). In conjunction with the absence of major replicative exonucleases, these G-quadruplexes could be involved in high levels of duplication formation observed in the mutants. It is also possible that regions of strong secondary structure, such as the *trnP*, which has been shown to be a barrier to POLG (30), may affect the formation of rearrangements. Alternatively, the recently identified LSP2 region in human mtDNA, which is within 5 bp of *trnP* (27), could also facilitate the formation of the observed duplications. However, this promoter region remains to be characterized in mice.

### The combined function of MGME1 and POLG EXO in mtDNA replication

Lastly, we determined that a double mutant *(Polg MM* and *Mgme1 KK*) is embryonically lethal. The reason for this became evident when double mutants were created in culture, as we found that mtDNA could not be maintained despite the presence of mitochondria. This showed that at least one of these exonucleases is necessary for successful mtDNA maintenance. This finding is intriguing because both *Mgme1 KK* and *Polg MM* models can maintain mtDNA. MGME1 has been found to process flaps formed by POLG, through strand displacement, at the end of replication (32). In fact, although MGME1 alone was found to be very poor at generating ligatable ends, it becomes very efficient in combination with POLG (6). POLG was found to “fix” the imprecise flaps or nicks generated by MGME1. The POLG 3′-5′ exonuclease trims the newly synthesized strand allowing newly created complementary flaps to reanneal to the template creating a ligatable nick instead. In WT cells and animals, it has been proposed that POLG and MGME1 work in concert to generate ligatable ends that allow for mtDNA replication (6) Having neither POLG EXO or MGME1, double mutant cells are likely unable to create any ligatable ends. Our findings validate these findings *in vivo* and suggest that this combined action of MGME1 and POLG EXO domain is critical for replication and other mitochondrial exonucleases, such as DNA2 or FEN1, cannot compensate for this combined deficiency. Based on the model proposed by the Falkenberg lab (6), we propose a model in which the concomitant loss of MGME1 would not allow for efficient formation of ligatable ends from these long flaps (Fig. 7*C*) leading to mtDNA depletion.

In conclusion, side-by-side comparison of mutants, MGME1 and POLG EXO showed similar roles in linearized mtDNA degradation after induced double strand mtDNA damage, while the granular analysis of the rearrangements breakpoints resulting from ablating either of these exonuclease activities suggests that MGME1 has a role in mtDNA replication beyond the previously established resolution of flaps at the end of replication. Lastly, we show that at least one of these DNA exonucleases is absolutely required for mtDNA maintenance. Our work highlights the interconnected role of these two enzymes in mtDNA degradation, replication, and maintenance.

## Experimental procedures

### Adenovirus preparation

Mito*Sca*I–HA was cloned into the pAdTrack5 adenoviral vector under the control of a CMV promoter. rAd–mito*Sca*I–HA adenovirus stocks were prepared by the Colorado State University Virus Core Facility. The adenovirus titers were estimated by OD260: rAd–mito*Sca*I–HA: 4 × 10^12^ particles/ml.

### Animal procedures

All mice procedures were performed according to a protocol approved by the University of Miami. Mice were housed in a virus/antigen-free facility at the University of Miami in a 12-h light/dark cycle at room temperature and fed ad libitum with a standard rodent diet. The *Polg* mutator mice (Jackson Laboratory: B6.129S7(Cg)-Polgtm1Prol/J, https://www.jax.org/strain/017341) contains an Asp->Ala in the second exonuclease domain (D257A), resulting in an inactive exonuclease. The *Mgme1* KO mice (Jackson Laboratory: https://www.jax.org/strain/030318) is missing exon 3, resulting in early termination and non-functional protein. We have confirmed the lack of MGME1 by Western blot.

#### rAd-mito*Sca*I-HA

Retro-orbital injections were performed on anesthetized mice at 28 to 30 days. *Mgme1KW*, *Mgme1 KK*, and C57WT Mice were injected with 2 × 10^11^ particles of rAd–mito*Sca*I–HA diluted to 50 μl in saline. Tissues were harvested 5 days after the retro-orbital injection. Anesthetized mice were transcardially perfused with ice-cold PBS and tissue was snap frozen immediately after collection. The liver was post-fixed overnight in 4% PFA, cryoprotected in 30% sucrose, and frozen in OCT using isopentane. Another, unfixed liver fragment was used for DNA purification and analyses. Non-injected mice were used as controls. Previous experiments showed that Adenovirus infection does not affect mtDNA integrity (10).

#### Tissues for DNA analyses

Tissues were collected from maximally aged female *Polg MM* (1Y)*, Mgme1 KK* (1Y3M), and from older C57WT (1Y5M) controls. Anesthetized mice were transcardially perfused with ice-cold PBS and tissue was snap frozen immediately after collection.

### Cell culture experiments

Primary cells from lung fibroblasts were generated in our laboratory from *Mgme1 KK, Mgme1KW*, *Polg MM, PolgMW* mice, and C57 littermate controls in high-glucose Dulbecco’s modified Eagle medium supplemented with 10% fetal bovine serum, 1 mM pyruvate, and 50 μg/ml uridine at 37 °C in an atmosphere of 5% CO2. Fibroblasts were immortalized by infection with a retrovirus expressing the E6 and E7 genes of type 16 Human Papilloma Virus carrying G418 resistance.

#### rAd-mito*Sca*I-HA

*Mgme1 KK, Mgme1KW*, *Polg MM, PolgMW*, and control fibroblasts were transduced in culture with 3 × 10^11^ particles of rAd–mitoScaI–HA. Viral particles were removed 1 day after transduction. Fibroblasts were collected at 1-, 2-, 5-, and 10-days post-transduction for DNA extraction.

#### Samples for DNA analyses

*Mgme1 KK*, *Polg MM,* and control fibroblasts were grown and collected at three different passage numbers.

### DNA extraction

Cells in culture were resuspended in RSB Buffer (10 mM Tris–HCl pH7.4, 10 mM NaCl, 25 mM EDTA pH 8.0) with Proteinase K (1 mg/ml) and 1% SDS and left to digest at 50 °C for 2 h. Tissues were pulverized using mortar, pestle, and liquid nitrogen, and resuspended in RSB buffer and left to digest at 37 °C overnight. DNA was extracted through phenol chloroform extraction and ethanol acetate precipitation.

### Quantitative PCR

Quantitative PCR reactions using TaqMan chemistry (PrimeTime Std qPCR Assay, IDT) were performed on a Bio-Rad CFX96/C1000 qPCR machine. We used the comparative ΔΔCt method to determine the relative quantity of mtDNA (33). The levels of different mtDNA species between nontransduced and transduced samples were determined by quantifying the levels of total mtDNA/genomic DNA (*Nd1*/*Actb*) or (D-loop/*Actb*). 5 to 10 ng/sample of total DNA, 2x IDT PrimeTime Gene Expression Master Mix (IDT# 1055770), the primers (final conc. 250 nM), and probes (final conc. 125 nM) below were mixed to a volume of 10 μl using the following cycling conditions (95 °C 3 min; [95 °C 5 s; 60 °C 30 s; 40 cycles]) and default imaging parameters. Primer/Probe Pairs.

#### mtDNA

IDT PrimeTime *Nd1* (1: GCCTGACCCATAGCCATAAT; 2: CGGCTGCGTATTCTACGTTA;

Probe: 56-FAM/TCTCAACCC/ZEN/TAGCAGAAACAAACCGG/3IABkFQ)

IDT PrimeTime D-loop (1: TCTCGATGGTATCGGGTCTAA; 2: CTTGACGGCTATGTTGATGAAA;

Probe: 5TET/AGCCCATGA/ZEN/CCAACATAACTGTGGT/3IABkFQ)

#### Genomic DNA

IDT PrimeTime β-Actin (*Actb)* (F: CTCCCTGGAGAAGAGCTATGA; R: CCAAGAAGGAAGGCTGGAAA; Probe: 5Cy5/TCATCACTATTGGCAACGAGCGGT/3IAbRQSp)

### Immunohistochemistry

Liver sections were cut to 18 μm thickness with a cryostat (Leica). For immunofluorescent staining, sections underwent antigen retrieval in 10 mM sodium citrate buffer. Sections were blocked for 1 h in 5% BSA in PBS at RT. Sections were incubated with primary antibody (Rat anti-HA 1:200 in 5% BSA (#ROAHAHA, Sigma)) for 16 h at 4 °C. Sections were then incubated with secondary antibody for 2 h at RT (Alexa-fluor anti-Rat/594 1:200 in 5% BSA (#A-11007, Molecular Probes)) and mounted with Vectashield hard set mounting medium with DAPI. Images were captured using a Zeiss LSM710 confocal microscope.

### Southern blot

Total DNA from fibroblasts was extracted using phenol: chloroform/isopropanol precipitation. Five micrograms of total DNA was digested with *Sac*I (NEB), separated on a 0.75% agarose gel, and transferred to a Zeta-Probe membrane (Bio-Rad). One template:1.7 kb (covering the D-loop) to generate probes to detect mtDNA and another template:1 kb (covering *18s* DNA) to generate probes to detect gDNA was amplified from genomic DNA from the cortex of a C57BL/6 J WT mouse. The following primers were used to amplify the mtDNA templates:1.7 kb (F: ATCCTCCGTGAAACCAACAA; R: GTCATGAAATCTTCTGGGTGTAGG) and the gDNA templates: 1 kb (F: CCCGGGGAGGTAGTGACGAAAAAT; R: CTGTGATGCCCTTAGATGTCCGG). Amplified DNA was purified, labeled with [α-32P] dCTP using Random Primed DNA Labeling kit (Roche), and cleaned with G-50 Sephadex quick spin columns (GE Healthcare). Detection and densitometric quantification of mtDNA signals was performed with the Cyclone Plus Phosphor Imager equipped with the Optiquant software (PerkinElmer).

## Next generation sequence analyses

### Whole genome sequencing

Whole genome sequencing (WGS) was used to analyze mouse heart mtDNA. Library construction and sequencing were performed at the Center for Genome Technology Sequencing Core (John P. Hussman Institute for Human Genomics, University of Miami Miller School of Medicine). Extracted DNA samples’ concentration was evaluated by fluorometric Qubit assays (Thermo Fisher Scientific) and for genome integrity by TapeStation (Agilent Technologies). Sequencing libraries were prepared with the TruSeq DNA PCR-free HT sample preparation kit from Illumina using 1 μg of total DNA according to manufacturer instructions. Briefly, DNA was fragmented with the aid of a Covaris LE220 focus acoustic sonicator to a target size of 350 bp. Blunt-end DNA fragments were size selected with AMPure bead purification (Beckman Coulter). A-base tailing was performed on the 3′ blunt ends, followed by adapter ligation and cleanup of the libraries using a bead-based kit. Final library fragment size was evaluated on the TapeStation (Agilent Technologies) and final molarity determined by qPCR with adapter-specific primers (Kapa Biosystems) on a Roche Light Cycler. Libraries were pooled for sequencing on a 10B flow cell on the Illumina NovaSeqX Plus to yield an average depth of 30 × per sample. FASTQ files were generated with the Illumina BCL2FASTQ algorithm and were used for downstream processing.

### WGS/mtDNA analysis

Raw FASTQ files were processed using a standard bioinformatics pipeline. Briefly, FASTQs were aligned to the (C57BL6) mtDNA (NC_005089.1) reference sequence using BWA-mem to avoid interference by nuclear pseudogenes. SNVs and short insertion-deletions variants (indels) were called using the GATK HaplotypeCaller.

### Mitochondrial DNA structural alterations

We applied Mitochondrial Structural Alterations, for identification, quantification, and circular plot visualization of both deletions and duplications in mtDNA as previously described (14). Briefly, raw reads underwent a global alignment against the mouse genome mm10 reference with Hisat2. Nuclear DNA aligned reads are discarded and the mtDNA reads are fed into a strict aligner (LAST). From the LAST results, MitoSALT further classifies the split/gapped reads determining if they are deletions or duplication events. Aligned bam files, bigwig files, indel designations, and R plots were taken directly from the MitoSALT analysis.

### GC content mtDNA map

GC content mtDNA map was generated using SnapGene (snapgene.com) software (Dotmatics).

### Western blot

Cell homogenates were prepared in PBS containing protease inhibitor mixture (Roche Diagnostics). Homogenates were snap frozen in liquid nitrogen, sonicated for 3 s, then centrifuged at 14,000*g* at 4 °C, and the supernatant collected. Protein concentration was determined by Lowry assay using the BCA kit (BioRad). Approximately 20 μg of protein were separated by SDS-PAGE in 7.5% acrylamide gels and transferred to PVDF membranes. Membranes were blocked with 5% non-fat milk in 0.1% Tween-20 in PBS and subsequently incubated in primary antibodies diluted in 0.5% milk in PBST. Rb anti-MGME1 1:500 (Rabbit polyclonal antisera obtained from Nils-Göran Larsson and Dusanka Milenkovic (7)) or Rb Anti-ATAD3 1:1000 (Proteintech 16610-1-AP) or Rb anti-TOM20 1:3000 (Santa Cruz Sc11415), all of which were validated with positive and negative controls. Secondary antibodies conjugated to horseradish peroxidase (Cell Signaling technologies) were used, and the reaction was developed by chemiluminescence using SuperSignal West reagent (Thermo Fisher Scientific) and exposed using a Chemidoc (Bio-Rad) with automatic optimal acquisition.

### Digital PCR

Qiagen QIAcuity One digital PCR system was used with 8.5 K partition 24 well QIAcuity Nanoplate (Qiagen #250011) or 8.5 K 96 well QIAcuity Nanoplate (Qiagen #250031). Up to 1 ng of DNA of total DNA from cells and tissue was used to characterize mtDNA fragmentation and copy number. DNA was mixed with 4x QIAcuity probe master mix (Qiagen# 250102); primers (final conc. 250 nM) and probes (final conc. 125 nM) below were mixed up to 12 μl using the following cycling conditions (95 °C 2 min; [95 °C 15 s; 55 °C 1 min; 40 cycles]) with default imaging parameters.

DNA isolated from cells and from tissue is first digested with *Sal*I RE (New England Biolabs, NEB #R3138S), a restriction enzyme that fragments the nuclear genome but does not target mtDNA. After digestion, DNA is diluted to arrive at a sample concentration suitable to examine molecular integrity and conduct copy number analysis. In effect, this minimizes the colocalization of different mtDNA fragments in the same partition. For cells, we used 0.5 to 1 ng per sample. For tissues, 0.1 to 0.4 ng per sample. We use the metric % positivity or the % of total partitions that have positive signal, to determine if the sample is diluted enough to determine the fragmentation profile by 2D dPCR. We used 20 to 80% positivity as the optimum (34). dPCR assays are as follows:

#### mtDNA

IDT PrimeTime *Nd1* (1: GCCTGACCCATAGCCATAAT; 2: CGGCTGCGTATTCTACGTTA; Probe: 56-FAM/TCTCAACCC/ZEN/TAGCAGAAACAAACCGG/3IABkFQ)

IDT PrimeTime *Nd4* (1: GAAGCAACCTTAATCCCAACAC; 2: AGCAGTGGAATAGAACCGATTAG; Probe: 5TET/ATGAGGGAA/ZEN/CCAAACTGAACGCCT/3IABkFQ)

IDT PrimeTime *CoxI* (1: TGGTGGTCTAACCGGAATTG; 2: AAACACTGCTCCCATTGATAGA; Probe: 5TET/TCCAACTCA/ZEN/TCCCTTGACATCGTGC/3IABkFQ)

IDT PrimeTime *Cytb* (1: CATGTCGGACGAGGCTTATATT, 2: CATGGAAGGACGTAGCCTATAAA; Probe: 56-FAM/TGTTCGCAG/ZEN/TCATAGCCACAGCA/3IABkFQ)

### TAG1 (200 bp)-IDT

AATTCGTACTTCGTTCAGAACTCACATTTTAACAACAGAGGACACATGCCCTACCTCCATGATCTACTGACGTCCCTGAGGCTGCAATACATGTAACGAGGCAGTATCCGCGGTAAGTCCTAGTGCAATGGCGGTTTTTTACCCTCGTCCTGGAGAAGAGGGGACGCCGGTGCAGTCATCACTAATGTGGAAATTGGGAG.

Forward ACATGTAACGAGGCAGTATCC (Sense)

Probe 56-FAM/ACCGCCA/ZEN/TTGCACTAGGACTTACC/3IABkFQ (AntiSense)

Reverse CTCCCAATTTCCACATTAGTGAT (AntiSense)

### TAG2(200 bp)-IDT

GGAGAATCTGTGCGGCAATGTCATTAATACATTTGAAACGCGCCGTACCGATGCTGAGCAAGTCAGTGCAGGCTCCCGTGTTAGGATAAGGGTAAACATACAAGTCGATAGAAGATGGGTAGGGGCCTTCAATTCATCCAACACTCTACGGCTCCTCCGAGAGCTAGTAGGGCACCCTGTAGTTGGAAGGGGAACTATTT.

Forward AGAATCTGTGCGGCAATGT (Sense)

Probe 5TET/CATTTGAAACGCGCCGTACCGATG (Sense)

Reverse TCGACTTGTATGTTTACCCTTATCC (AntiSense)

#### Nuclear DNA

IDT Primetime *18s* (1: CGTCTGCCCTATCAACTTT; 2: CCTCGAAAGAGTCCTGTATTG, Probe: 5Cy5/AGAAACGGCTACCACATCC/3IAbRQSp/)

#### Construction of synthetic DNA molecule

The synthetic molecule was built using restriction enzyme digestions, gene blocks, and Takara Bio’s In-Fusion Snap Assembly Kit.

### Generation of double mutant (*Polg MM*/*Mgme1 KK*)

Using Integrated DNA Technologies’ Custom Alt-R CRISPR-Cas9 System, we created two different Ribonucleoprotein (RNP) complexes using 2 crRNA (CRISPR RNA) all targeting exon 2 of *Mgme1* in combination with 1 tracrRNA (Transactivating CRISPR RNA). gRNA1: CTAAACGAGAGTACTTCGCT and gRNA2: CTGAAGGATGCGGGTCACAC. Using the Neon transfection system, we introduced the RNP complex into *Polg MM* lung fibroblasts (2 separate transfections, 1 per RNP). Lastly, using Synthego’s Inference of CRISPR Edits tool, we screened resulting pools of cells by Sanger sequencing. Using dilution cloning, we were able to isolate two clones (1g1e11 and 2g1c11) which lacked MGME1 as confirmed by Western blot (Fig. 6) and then sequence aligned these clones to the parental *Polg MM* cell line (Figs. 6 and S9) to show divergent regions.

### Statistics and reproducibility

Sample sizes were determined based on previous publications, and independent biological replicates range from three to four for all experimental modalities used in this study. No data were excluded from the analysis. A few replicates are missing due to a failure of acquisition of the image after dPCR. The experiments were not randomized. The investigators were not blinded to allocation during experiments and outcome assessment.

## Data availability

The data needed to evaluate the conclusions in the paper are present in the paper and/or the Supplementary Materials. The NGS data was deposited as BioProject PRJNA1038786.

## Supporting information

This article contains supporting information (14).

## Conflict of interest

The authors declare that they have no conflicts of interest with the contents of this article.

## References

[1] Falkenberg M., Gustafsson C.M. (2020). Mammalian mitochondrial DNA replication and mechanisms of deletion formation. Crit. Rev. Biochem. Mol. Biol..

[2] Fontana G.A., Gahlon H.L. (2020). Mechanisms of replication and repair in mitochondrial DNA deletion formation. Nucleic Acids Res..

[3] Rahman S., Copeland W.C. (2019). POLG-related disorders and their neurological manifestations. Nat. Rev. Neurol..

[4] Kujoth G.C., Hiona A., Pugh T.D., Someya S., Panzer K., Wohlgemuth S.E. (2005). Mitochondrial DNA mutations, oxidative stress, and apoptosis in mammalian aging. Science.

[5] Trifunovic A., Wredenberg A., Falkenberg M., Spelbrink J.N., Rovio A.T., Bruder C.E. (2004). Premature ageing in mice expressing defective mitochondrial DNA polymerase. Nature.

[6] Uhler J.P., Thorn C., Nicholls T.J., Matic S., Milenkovic D., Gustafsson C.M. (2016). MGME1 processes flaps into ligatable nicks in concert with DNA polymerase gamma during mtDNA replication. Nucleic Acids Res..

[7] Matic S., Jiang M., Nicholls T.J., Uhler J.P., Dirksen-Schwanenland C., Polosa P.L. (2018). Mice lacking the mitochondrial exonuclease MGME1 accumulate mtDNA deletions without developing progeria. Nat. Commun..

[8] Milenkovic D., Sanz-Moreno A., Calzada-Wack J., Rathkolb B., Veronica Amarie O., Gerlini R. (2022). Mice lacking the mitochondrial exonuclease MGME1 develop inflammatory kidney disease with glomerular dysfunction. PLoS Genet..

[9] Korhonen J.A., Pham X.H., Pellegrini M., Falkenberg M. (2004). Reconstitution of a minimal mtDNA replisome *in vitro*. EMBO J..

[10] Nissanka N., Bacman S.R., Plastini M.J., Moraes C.T. (2018). The mitochondrial DNA polymerase gamma degrades linear DNA fragments precluding the formation of deletions. Nat. Commun..

[11] Peeva V., Blei D., Trombly G., Corsi S., Szukszto M.J., Rebelo-Guiomar P. (2018). Linear mitochondrial DNA is rapidly degraded by components of the replication machinery. Nat. Commun..

[12] Kornblum C., Nicholls T.J., Haack T.B., Scholer S., Peeva V., Danhauser K. (2013). Loss-of-function mutations in MGME1 impair mtDNA replication and cause multisystemic mitochondrial disease. Nat. Genet..

[13] Moretton A., Morel F., Macao B., Lachaume P., Ishak L., Lefebvre M. (2017). Selective mitochondrial DNA degradation following double-strand breaks. PLoS One.

[14] Basu S., Xie X., Uhler J.P., Hedberg-Oldfors C., Milenkovic D., Baris O.R. (2020). Accurate mapping of mitochondrial DNA deletions and duplications using deep sequencing. PLoS Genet..

[15] Fragkoulis G., Hangas A., Fekete Z., Michell C., Moraes C.T., Willcox S. (2024). Linear DNA-driven recombination in mammalian mitochondria. Nucleic Acids Res..

[16] Bruni F., Lightowlers R.N., Chrzanowska-Lightowlers Z.M. (2017). Human mitochondrial nucleases. FEBS J..

[17] Nicholls T.J., Zsurka G., Peeva V., Scholer S., Szczesny R.J., Cysewski D. (2014). Linear mtDNA fragments and unusual mtDNA rearrangements associated with pathological deficiency of MGME1 exonuclease. Hum. Mol. Genet..

[18] Williams S.L., Huang J., Edwards Y.J., Ulloa R.H., Dillon L.M., Prolla T.A. (2010). The mtDNA mutation spectrum of the progeroid Polg mutator mouse includes abundant control region multimers. Cell Metab..

[19] Ameur A., Stewart J.B., Freyer C., Hagstrom E., Ingman M., Larsson N.G. (2011). Ultra-deep sequencing of mouse mitochondrial DNA: mutational patterns and their origins. PLoS Genet..

[20] Gustafsson C.M., Falkenberg M., Larsson N.G. (2016). Maintenance and expression of mammalian mitochondrial DNA. Annu. Rev. Biochem..

[21] Lee H.C., Pang C.Y., Hsu H.S., Wei Y.H. (1994). Ageing-associated tandem duplications in the D-loop of mitochondrial DNA of human muscle. FEBS Lett..

[22] Wei Y.H., Pang C.Y., You B.J., Lee H.C. (1996). Tandem duplications and large-scale deletions of mitochondrial DNA are early molecular events of human aging process. Ann. N. Y Acad. Sci..

[23] Williams S.L., Mash D.C., Zuchner S., Moraes C.T. (2013). Somatic mtDNA mutation spectra in the aging human putamen. PLoS Genet..

[24] Dahal S., Dubey S., Raghavan S.C. (2018). Homologous recombination-mediated repair of DNA double-strand breaks operates in mammalian mitochondria. Cell. Mol. Life Sci..

[25] Klucnika A., Mu P., Jezek J., McCormack M., Di Y., Bradshaw C.R. (2023). REC drives recombination to repair double-strand breaks in animal mtDNA. J. Cell Biol..

[26] Rong Z., Tu P., Xu P., Sun Y., Yu F., Tu N. (2021). The mitochondrial Response to DNA Damage. Front. Cell Dev. Biol..

[27] Tan B.G., Mutti C.D., Shi Y., Xie X., Zhu X., Silva-Pinheiro P. (2022). The human mitochondrial genome contains a second light strand promoter. Mol. Cell.

[28] Falabella M., Kolesar J.E., Wallace C., de Jesus D., Sun L., Taguchi Y.V. (2019). G-quadruplex dynamics contribute to regulation of mitochondrial gene expression. Sci. Rep..

[29] Doimo M., Chaudhari N., Abrahamsson S., L'Hote V., Nguyen T.V.H., Berner A. (2023). Enhanced mitochondrial G-quadruplex formation impedes replication fork progression leading to mtDNA loss in human cells. Nucleic Acids Res..

[30] Sullivan E.D., Longley M.J., Copeland W.C. (2020). Polymerase gamma efficiently replicates through many natural template barriers but stalls at the HSP1 quadruplex. J. Biol. Chem..

[31] Kiktev D.A., Sheng Z., Lobachev K.S., Petes T.D. (2018). GC content elevates mutation and recombination rates in the yeast Saccharomyces cerevisiae. Proc. Natl. Acad. Sci. U. S. A..

[32] He Q., Shumate C.K., White M.A., Molineux I.J., Yin Y.W. (2013). Exonuclease of human DNA polymerase gamma disengages its strand displacement function. Mitochondrion.

[33] Schmittgen T.D., Livak K.J. (2008). Analyzing real-time PCR data by the comparative C(T) method. Nat. Protoc..

[34] Majumdar N., Wessel T., Marks J. (2015). Digital PCR modeling for maximal sensitivity, dynamic range and measurement precision. PLoS One.

