# Supplementary material for: Absence of both MGME1 and POLG EXO abolishes mtDNA whereas absence of either creates unique mtDNA duplications
Source: J Biol Chem. 2024 Mar 1;300(4):107128. doi: 10.1016/j.jbc.2024.107128 (PMC11002302; doi:10.1016/j.jbc.2024.107128)
Supplement: Supporting Figures [file mmc1.pdf]

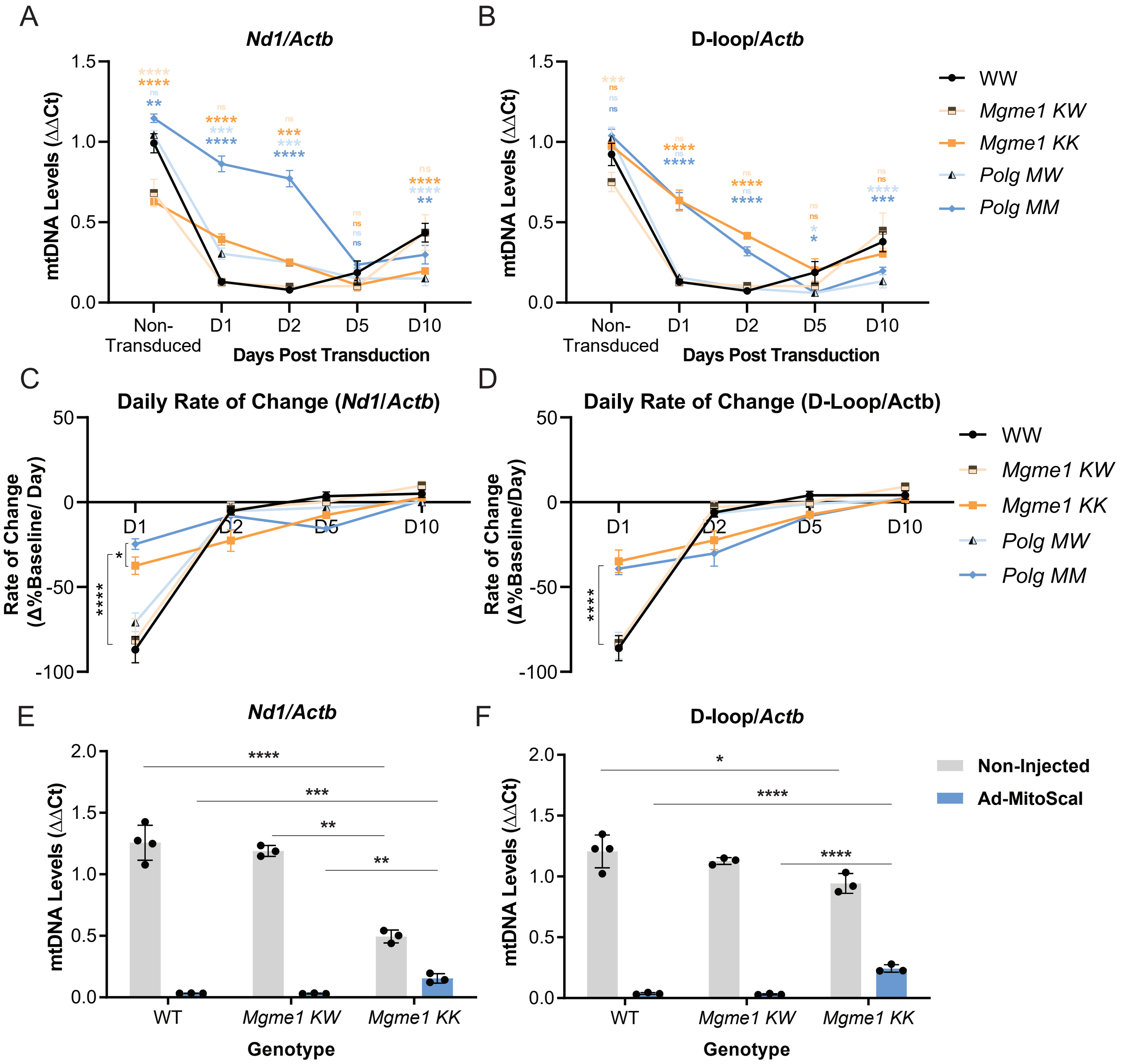

Supplemental Figure S1. MtDNA levels quantified via qPCR (ΔΔCT) after induced double strand break
A-B) The same samples were used to generate the graphics in Fig1A,B. The graphics shown here are the standard ΔΔ CT quantification method for qPCR. Points are an average (n=3) and error bars are SD. **C-D)** Daily rate of change in mtDNA levels post transduction with rAd-mitoScaI-HA. Quantified as the change in mtDNA levels divided by the number of days in between the measurements (Δ %Baseline). Points are an average (n=3), and error bars are SD. Comparisons shown highlight the difference among the homozgyous mutants and between the homozygous mutants and the rest of the cell models. Baseline levels of *Nd1* and D-Loop can be found in panels A-B. **E-F)** mtDNA levels were quantified via qPCR (ΔΔCT). Same samples were used to generate Fig1D,F. Bar graphs show the mean n=3-4 animals depending on the group, and error bars are SD. A two-way ANOVA was performed to analyze the effect of genotype and time elapsed post-transduction on daily rate of change in mtDNA levels. A one-way ANOVA was performed to compare the effect of genotype on mtDNA levels in mice expressing mitochondrial targeted endonuclease. Another one-way ANOVA was performed to compare the effect of genotype on mtDNA levels in mice not expressing mitochondrial targeted endonuclease. Multiple comparison analysis with Tukey correction was used to determine the statistically significant differences depicted on the graphs. (ns=not significant, *p<0.05, **p<0.01, ***p<0.005, ****p<0.0001).

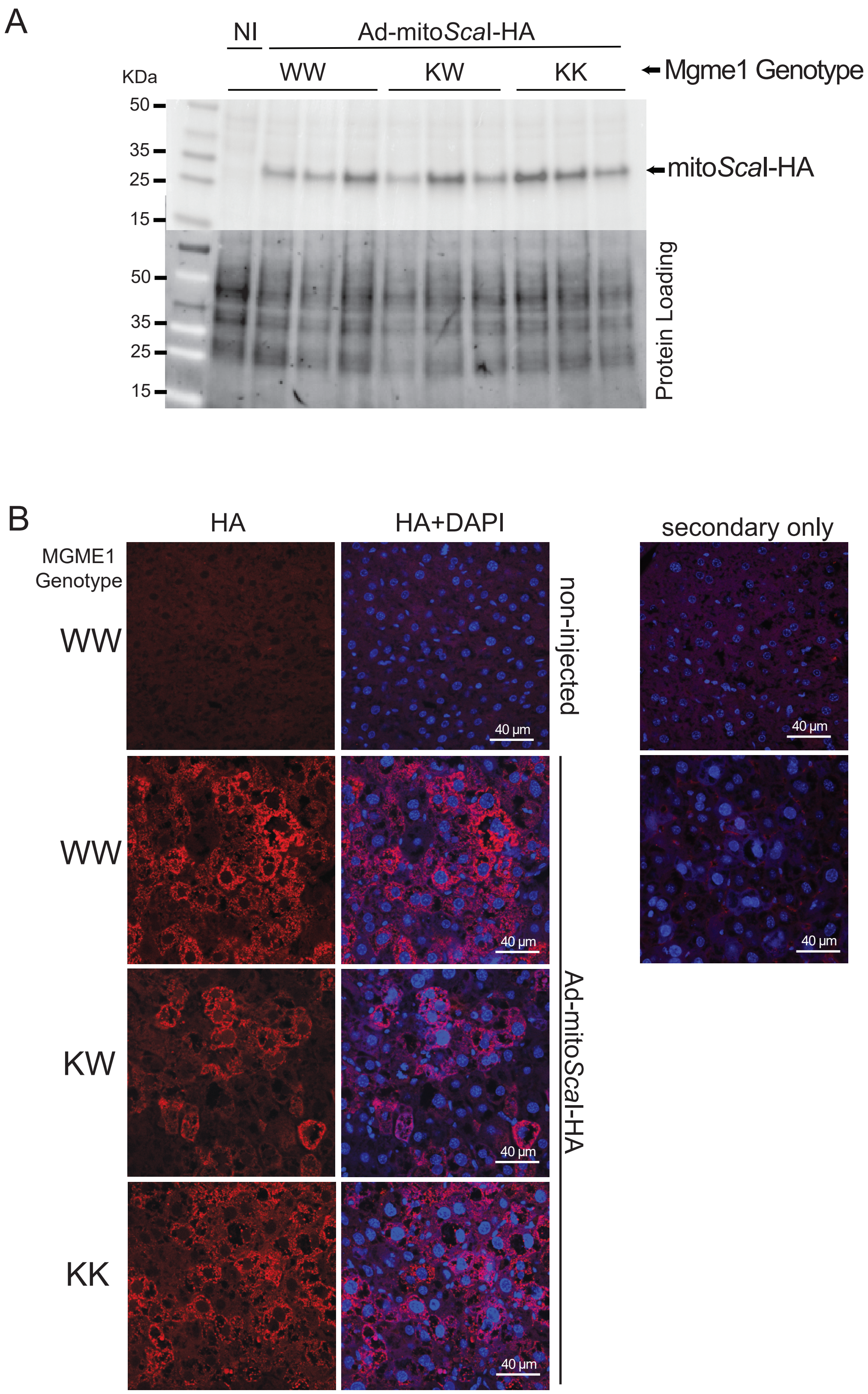

Supplemental Figure S2. Mice systemically injected with Ad-MitoScal express MitoScal-HA in the liver.

Mgme1 KK and heterozygous (KW) and homozygous (WW) controls (KW) were retroorbitally injected with recombinant Adenovirus expressing rAd-mitoScal-HA. Liver was analyzed 5 days after injection. **A)** Total liver protein lysate was analyzed by western blot (20 µg/ well) and labeled with anti-HA antibody. n=3 in each genotype injected with rAd-mitoScal-HA . NI = Not Injected. **B)** Liver was sectioned into 18 µm slices and stained with anti-HA AlexaFluor^{TM} antibody. NI = Not Injected.

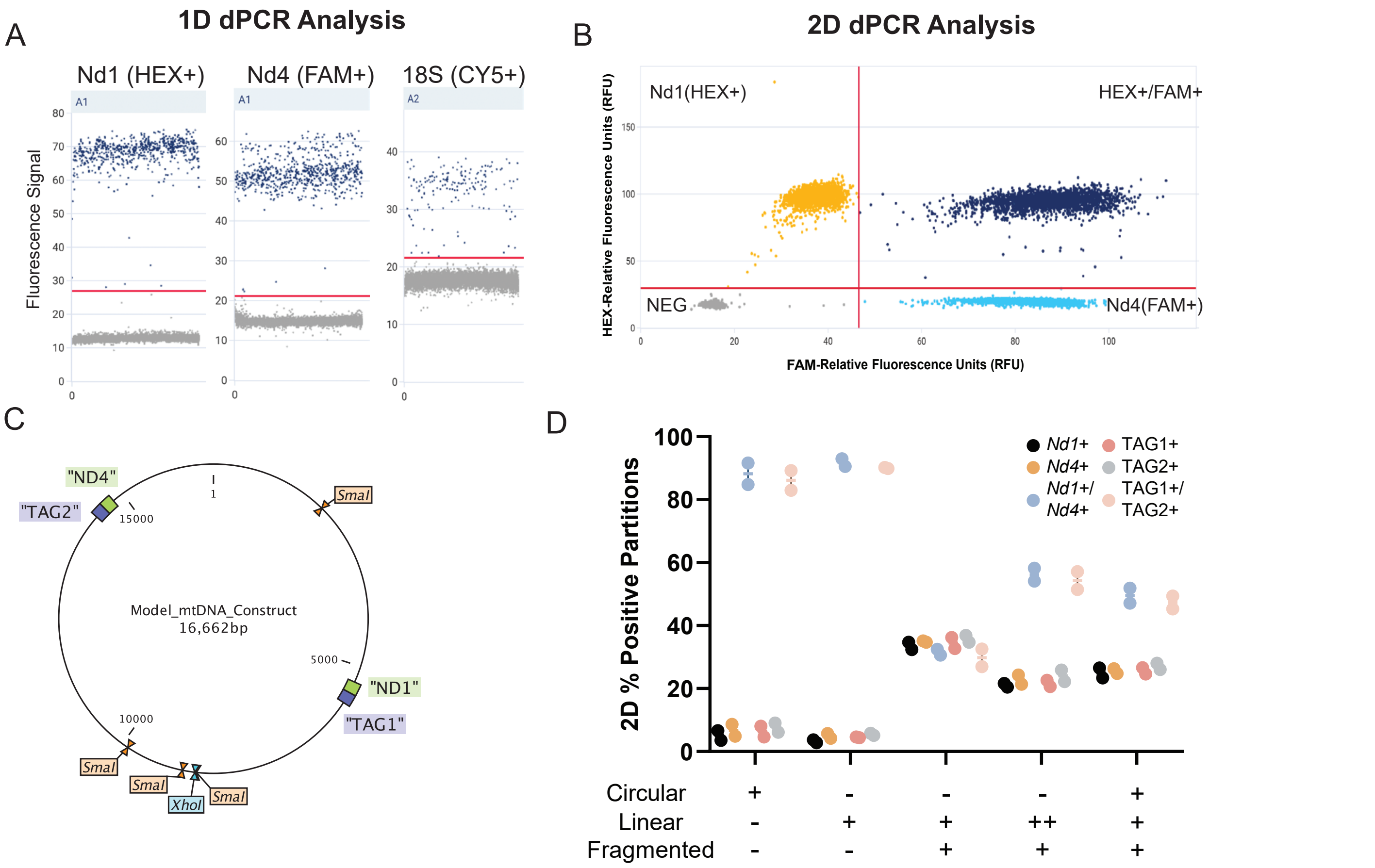

Supplemental Figure S3. Defining structural patterns by 1D and 2D dPCR analyses.
A) Example of the dPCR raw data (1D) used for copy number analysis of *Nd1*, *Nd4*, and *18s*. B) Example of a 2D plot generated by Qiagen's QIAcuity Software Suite showing the relative fluorescence units (RFU) for each partition for two Taqman assays (i.e., FAM, HEX) on a scatter plot. Each dot represents a partition. Using the total count of partitions with signal (100%) we could determine the 2D % Positive Partitions (2D% PP) of SPPs and DPPs. C) Map of relevant targets on an artificial mtDNA model molecule. D) Fragmentation profile of model DNA molecules. Circular, linearized but not fragmented, and fragmented model mtDNA were mixed and analyzed using dPCR 2D plot analysis. The relative volumetric components for each condition are depicted by the number of + (e.g. the first column only has 1 part circular model DNA, while the second column has 1 part linear).

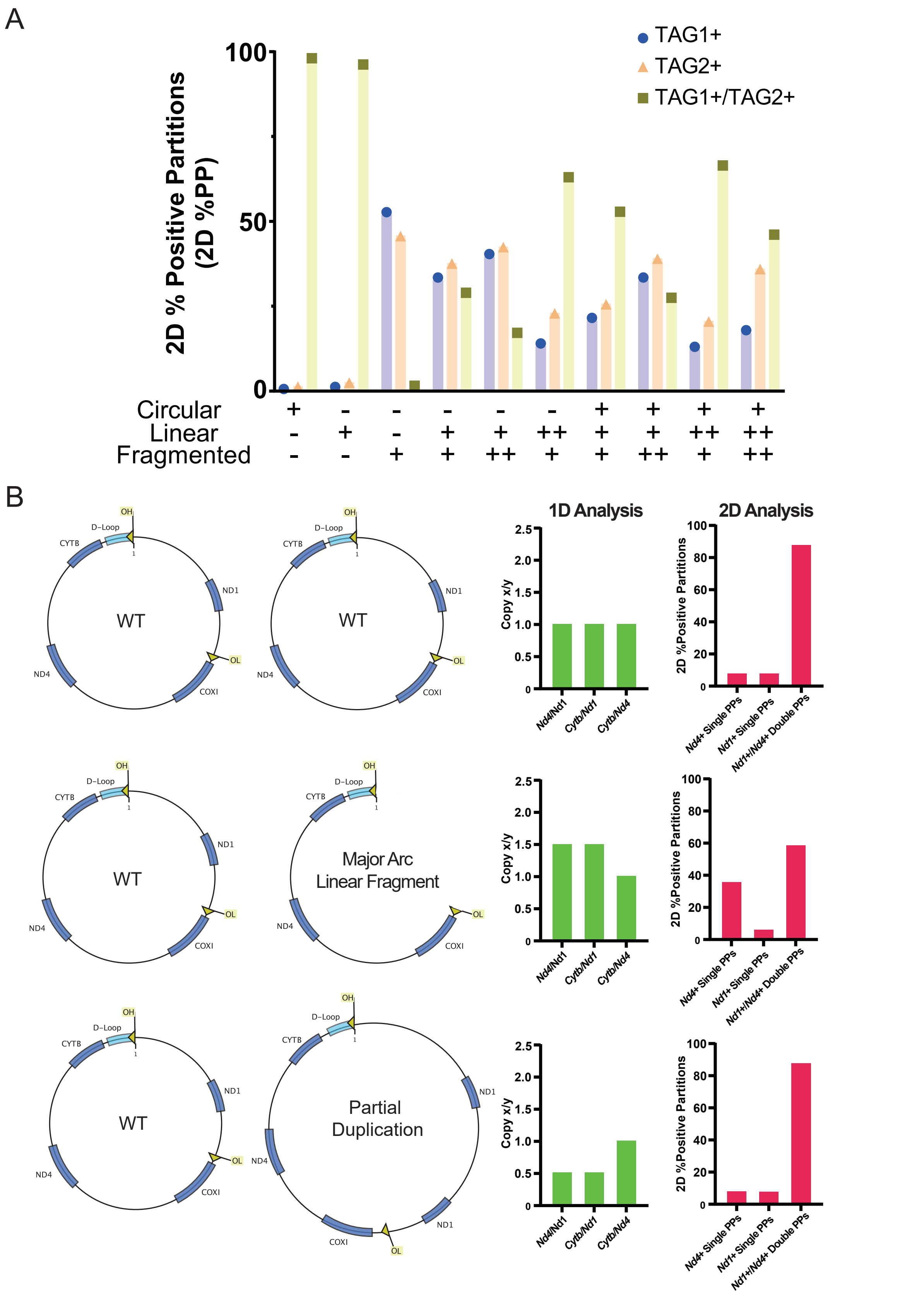

Supplemental Figure S4. Changes in 2D% PP reflect changes in DNA structure
A) Large array of fragmentation profiles of model DNA molecules using TAG1 and TAG2. **B)** Changes in copy number (1D analysis) and in 2D %PP for SPPs and DPPs (2D analysis) reflect the different DNA structures present in the sample.

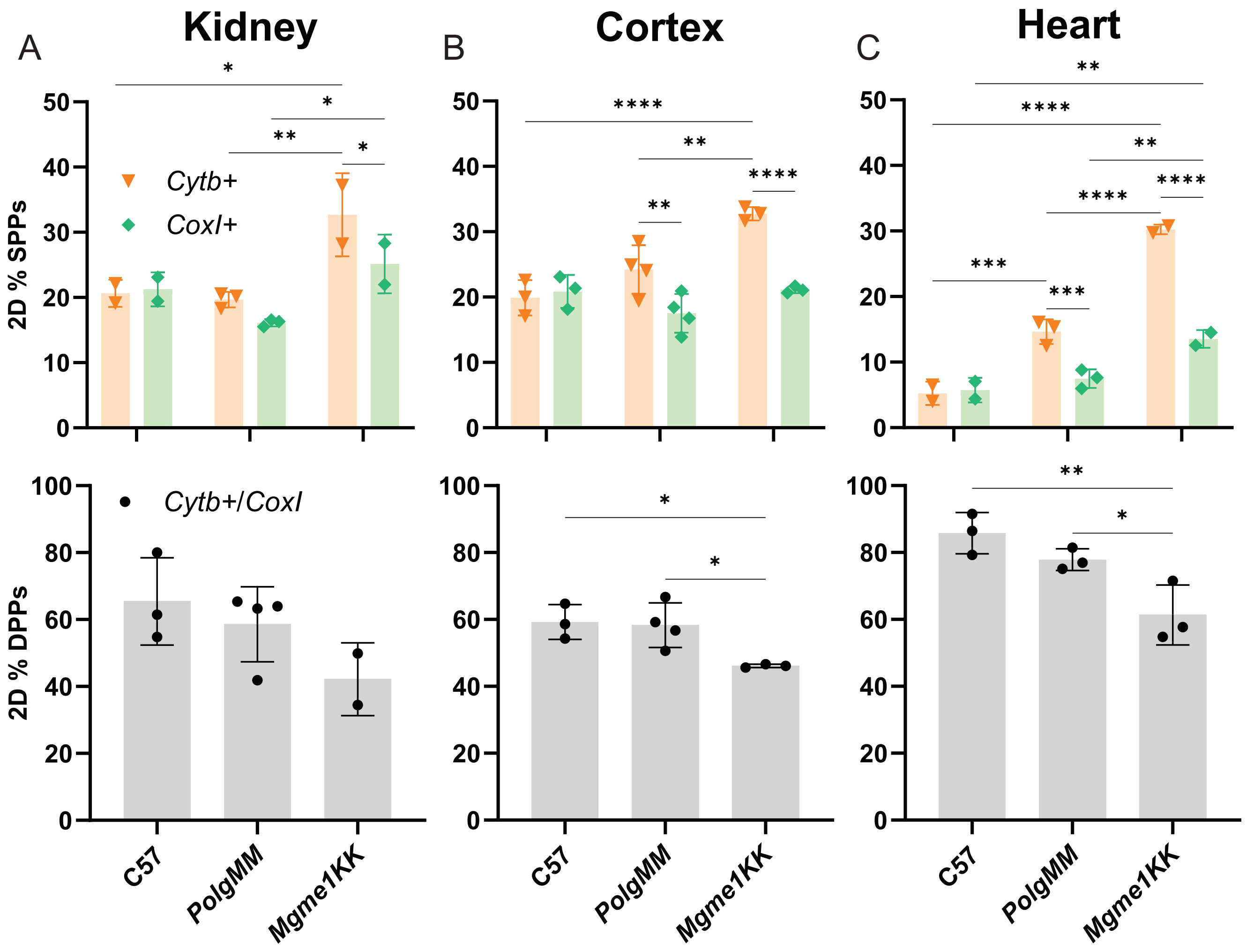

Supplemental Figure S5. *Mgme1KK* and *PolgMM* animals have increased levels of fragmented mtDNA
A-C) 2D %PP was measured in *Cytb*+ and *CoxI*+ SPPs (Top Row) and *Cytb*+/*Cox*I+ DPPs (Bottom Row) using 2D plot dPCR analysis on C57WT, *PolgMM, Mgme1KK* animal tissue (n=3-4 per group). Each point represents a different animal, the bar is the average 2D %PP of animals in that group, error bars are SD. Two-way ANOVAs were performed to compare the effect of genotype and mtDNA target on 2D %PP of SPPs depicted. One-way ANOVAs were performed to compare the effect of the genotype on 2D %PP for DPPs depicted. Multiple comparison analysis with Tukey correction was done to determine the statistically significant differences depicted on the graphs (*p<0.05, **p<0.01). (*p<0.05, **p<0.01, ***p<0.005, ****p<0.0001).

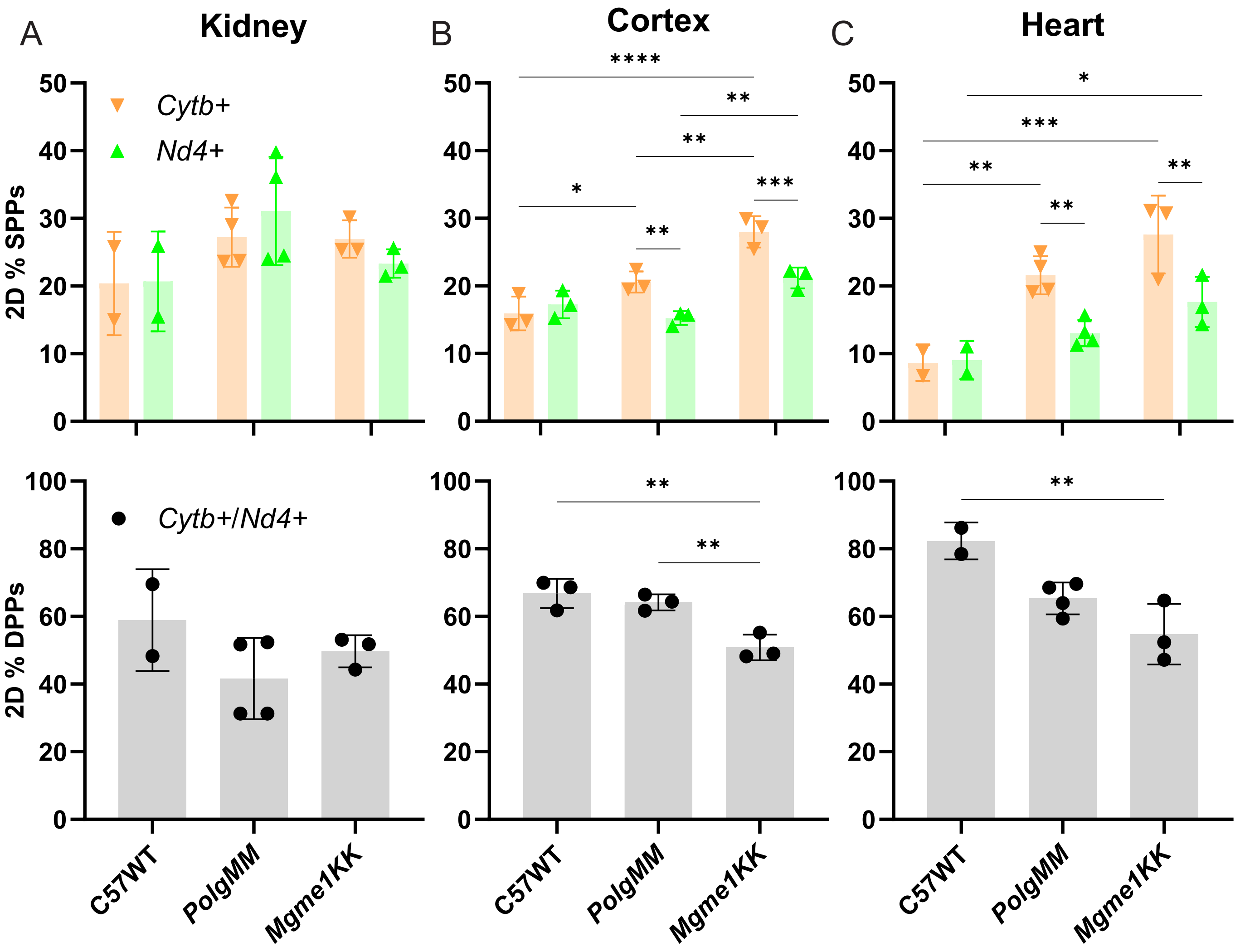

Supplemental Figure S6. *Mgme1KK* and *PolgMM* animals have increased levels of fragmented mtDNA
A-C) 2D %PP was measured in *Cytb*+ and *Nd4*+ SPPs (Top Row) and *Cytb+/Nd4*+ (Bottom Row) DPPs using 2D plot dPCR analysis using animal tissue (n=3-4 per group). Each point represents a different animal, the bar is the average 2D %PP of all animals in that group, error bars are SD. (*p<0.05, **p<0.01). (*p<0.05, **p<0.01, ***p<0.005, ****p<0.0001).

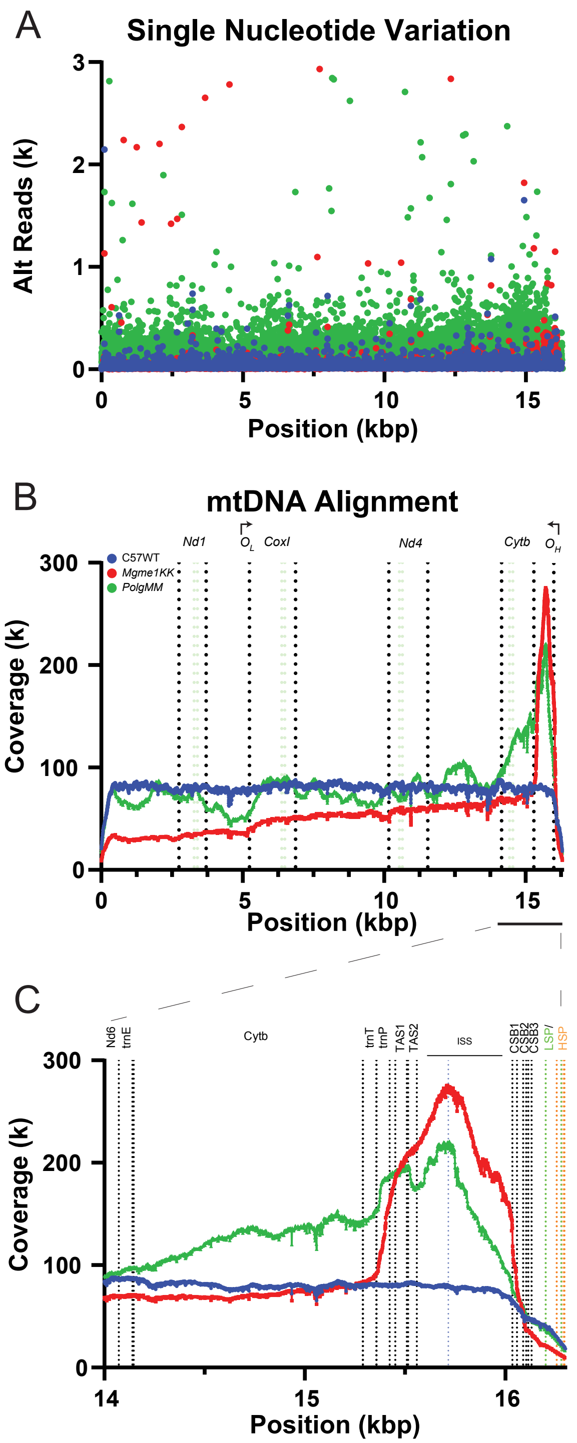

Supplemental Figure S7. Single nucleotide variant (SNV) analysis and *Mgme1 KK* and *Polg MM* mouse heart NGS alignment. A) SNV analysis shows alternative reads for each position on the murine mtDNA sequence using the standard reference (NC_005089.1 C57BL/6J). **B)** Next generation sequence (NGS) alignment of DNA from the heart of C57WT, *Mgme1 KK* and *Polg MM* animals, using NC_005089.1, C57BL/6J, as a reference (N=1). Genes used to measure mtDNA copies via dPCR are shown (black dashed lines) with the primer amplified regions (green dashed lines). Additionally, the origin of replication for both the heavy strand (O_H) and the light strand (O_L) and the direction of replication is noted. **C)** A closer look at the NGS shown in pane A with higher resolution mapping of of the control region.

♂ *Polg MW/Mgme1 KW*
x
♀ *Polg MW/Mgme1 KW*

POLG MGME1	expected	actual
WW WW	6-7	14
WW KW	13-14	22
WW KK	6-7	4
MW WW	13-14	20
MW KW	27-28	33
MW KK	13-14	0
MM WW	6-7	12
MM KW	13-14	4
MM KK	6-7	0
# genotyped		109

♂ *Polg MM/Mgme1 KW*
x
♀ *Polg WW/Mgme1 KK*

POLG MGME1	expected	actual
MW KW	3	6
MW KK	3	0
# genotyped		6

♂ *Polg MM/Mgme1 KW*
x
♀ *Polg MW/Mgme1 KW*

POLG MGME1	expected	actual
MW WW	1-2	1
MW KW	3-4	7
MW KK	1-2	0
MM WW	1-2	4
MM KW	3-4	2
MM KK	1-2	0
# genotyped		14

Supplemental Figure S8. Animal crosses attempted to generate a *PolgMM / Mgme1KK* animal. Three different types of crosses were performed to obtained double mutant mice (*PolgMM / Mgme1KK*). The parent males and female genotypes are shown on top of each table. The tables show the expected pup genotype results (based on Punnett square calculations) of the pairings vs the actual pup genotype. Cells in orange emphasize the lack of double mutant mice, even with only one mutator allele.

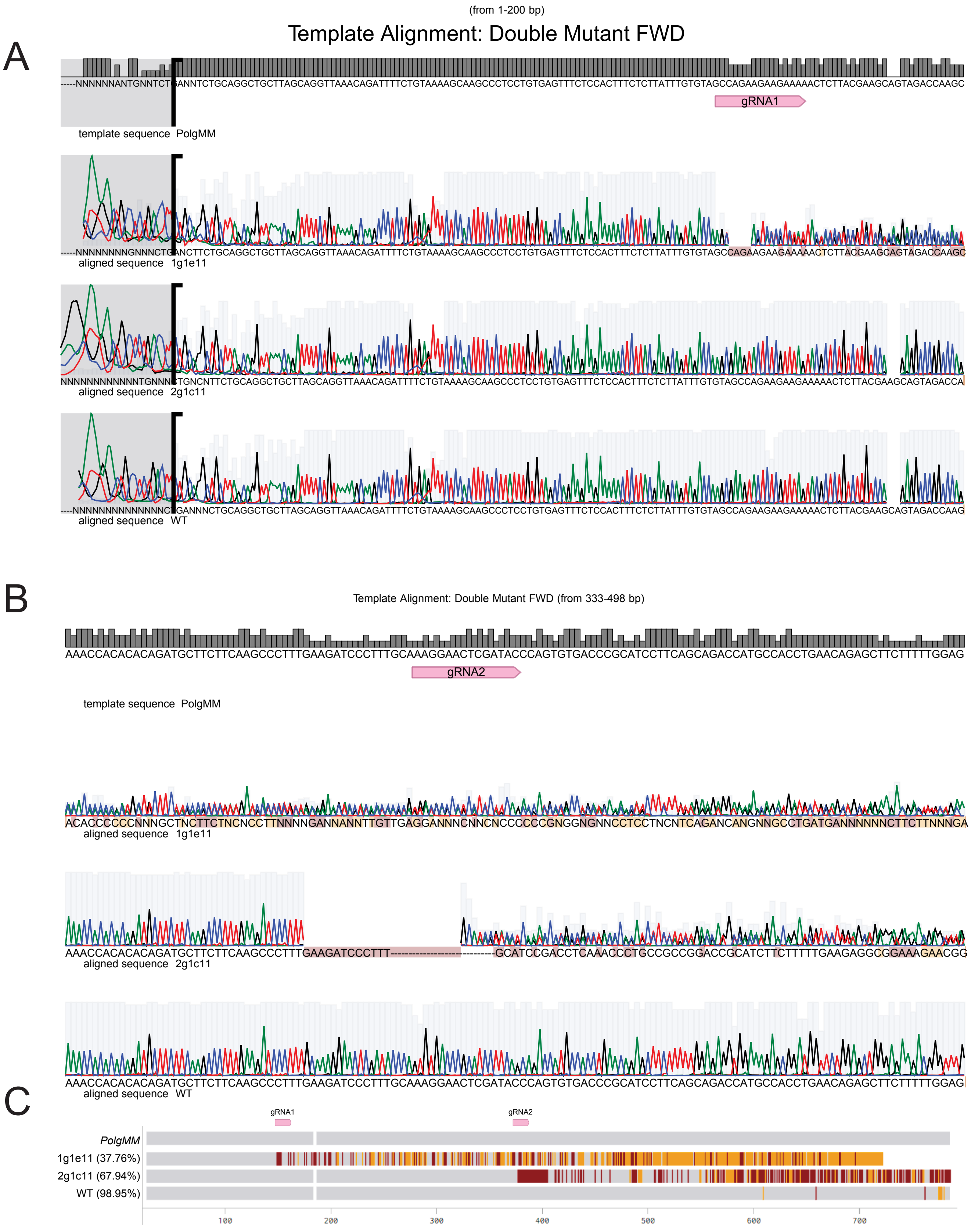

Supplemental figure S9. Sanger sequence alignment of double mutant clones. Comparing *Polg MM/Mgme1 KK* to the parental cell line *PolgMM* (top trace, reference) and WT (bottom trace). A) Aligment of 1g1e11 which was generated using guide RNA1. B) Alignment of 2g1c11 which was generated using guide RNA 2. C) ~800 bp sequence alignment of each cell line showing divergent regions.
