# Supplementary material for: Absence of both MGME1 and POLG EXO abolishes mtDNA whereas absence of either creates unique mtDNA duplications
Source: J Biol Chem. 2024 Mar 1;300(4):107128. doi: 10.1016/j.jbc.2024.107128 (PMC11002302; doi:10.1016/j.jbc.2024.107128)
Supplement: Supporting Table S1 [file mmc2.pdf]

Duplication Breakpoint			
Genotype	Start	End	Mechanism
Mgme1KK	trnP	CSB	Strand Displacement
	CSB	12sRNA	Recombination?
	trnP	LSP/HSP	Strand Displacement
	trnP	12sRNA	Recombination?
	CSB	LSP/HSP	Strand Displacement
	Cont.	Cont.	Replication Slippage
PolgMM	trnP	ISR	Strand Displacement

Supplementary Table 1. Start and end breakpoint regions found via mitoSALT analysis Most frequent start and end pairs were noted in this table in descending order of frequency, along with the predicted mechanism of formation.
